# Recognizing Skateboard and Kickboard Commuting Behaviors Using Activity Trackers: Feasibility Study Using Machine Learning Approaches

**DOI:** 10.2196/71969

**Published:** 2025-08-29

**Authors:** Nathanael Aubert-Kato, Hitomi Hatori, Arisa Orihara, Takashi Nakagata, Yuji Ohta, Julien Tripette

**Affiliations:** 1 Department of Computer Science, Ochanomizu University, Tokyo, Japan; 2 Center for Interdisciplinary AI and Data Science, Ochanomizu University, 2-1-1 Otsuka, Bunkyo District, Tokyo, 112-8610, Japan, 81 359783032 ext 3032; 3 Department of Human-Environmental Sciences, Ochanomizu University, Tokyo, Japan; 4 Center for Physical Activity Research, National Institutes of Biomedical Innovation, Health and Nutrition, Settsu, Japan; 5 Laboratory of Gut Microbiome for Health, Microbial Research Center for Health and Medicine, National Institutes of Biomedical Innovation, Health and Nutrition, Settsu, Japan

**Keywords:** physical activity, activity recognition, wearable sensor, skateboard, machine learning

## Abstract

**Background:**

Active commuting, such as skateboarding and kickboarding, is gaining popularity as an alternative to traditional modes of transportation such as walking and cycling. However, current activity trackers and smartphones, which rely on accelerometer data, are primarily designed to recognize symmetrical locomotive activities (eg, walking and running) and may struggle to accurately identify the unique push-push-glide motion patterns of skateboarding and kickboarding.

**Objective:**

The primary objective of this study was to evaluate the feasibility of classifying skateboard and kickboard commuting behaviors using data from wearable sensors and smartphones. A secondary objective was to identify the most important sensor-derived features for accurate activity recognition.

**Methods:**

Ten participants (4 women and 6 men; aged 12‐55 y) performed 9 activities, including skateboarding, kickboarding, walking, running, bicycling, ascending stairs, descending stairs, sitting, and standing. Data were collected using wearable sensors (accelerometer, gyroscope, and barometer) placed on the wrist and the hip, as well as in the pocket to replicate the sensing characteristics of commercial activity trackers and smartphones. The signal processing approach included the extraction of 211 features from 10- and 20-second sliding windows. Random forest classifiers were trained to perform multiclass and binary classifications, including distinguishing skateboarding and kickboarding from other activities.

**Results:**

Wrist-worn sensor configurations achieved the highest balanced accuracies for multiclass classification (range 84%‐88%). Skateboarding and kickboarding were identified with high sensitivity, ranging from 93% to 99% and 97% to 99%, respectively. Hip and pocket sensor configurations showed lower performance, particularly in distinguishing skateboarding (range 49%‐58% sensitivity) from kickboarding (78% sensitivity). Binary classification models grouping skateboarding and kickboarding into a push-push-glide superclass achieved high accuracies (range 91%‐95%). Key features for classification included low- and high-frequency accelerometer signals, as well as roll-pitch-yaw angles.

**Conclusions:**

This study demonstrates the feasibility of recognizing skateboard and kickboard commuting behaviors using wearable sensors, particularly wrist-worn devices. While hip and pocket sensors showed limitations in differentiating these activities, the broader push-push-glide classification achieved acceptable accuracy, suggesting its potential for integration into activity tracker software. Future research should explore sensor fusion approaches to further enhance recognition performance and address the question of energy expenditure estimation.

## Introduction

### Background

Active commuting is increasingly recognized as an effective way to encourage healthy habits among individuals with limited opportunities for regular physical activity (PA) [1,2]. According to a systematic review and meta-analysis by Dinu et al [3], active commuters experience an 8% reduction in mortality risk and a 9% to 30% lower risk of various noncommunicable diseases.

While walking and cycling remain the most common forms of active commuting [4], alternative nonmotorized transportation modes such as skateboarding and kickboarding are gaining popularity [5]. Often described as “fun, faster than walking, and more convenient than a bike” [6], skateboards and similar devices are involved in >30,000 multimodal trips per day in Los Angeles, California [5]. In Portland, Oregon, at least 1 skateboarder passes through 79% of the intersections of the city. Skateboarding is particularly popular among young adults, with mode shares reaching up to 7% on university campuses in California. Outside the United States, Ward et al [7] reported that 7% of surveyed teenagers in New Zealand used skateboarding as a mode of transport. Skateboarding, specifically in a cruising context, can reach intensities as high as 8.5 metabolic equivalents of task (METs), as compared to 3.5 METs for walking, 6.8‐9.3 METs for bicycling, and 6.8 METs for e-bicycling [8,9]. The similar motion pattern—characterized by several successive kicking or pushing actions followed by a gliding phase, hereafter referred to as the “push-push-glide” pattern—suggests that the intensity of kickboarding may be comparable to that of skateboarding. In a treadmill-based laboratory study, Kijima et al [10] found that kickboarding elicited energy expenditures ranging from 3.9 to 5.0 METs. Collectively, these findings indicate that kickboard and skateboard commuting may be viable strategies for individuals seeking to increase their daily PA.

Activity trackers are wearable devices equipped with sensors that evaluate PA and provide users with quantitative feedback, such as daily energy expenditure and step counts. These devices are commonly worn on the wrist and the hip or placed in the pocket. Nowadays, many smartphone apps leverage the sensing capabilities of smartphones to perform similar PA assessment. While modern activity trackers and smartphones may feature a variety of advanced sensing technologies, their core evaluation method typically relies on processing accelerometer data to estimate PA intensity [11].

Although algorithms developed by consumer-grade activity tracker manufacturers are often proprietary, several published studies using devices that provide access to raw data have aimed to develop accurate software tools for estimating energy expenditure, shedding light on the data processing flow of activity tracker algorithms [12-18]. They generally use sliding analytic windows, ranging in length from 1 to 60 seconds, and involve 4 successive operations. First, signals are smoothed to filter out noise-unrelated human movement, typically within the 0.25‐10.00 Hz frequency range. Second, signal features are extracted in both the time and frequency domains [16]. Third, the extracted data are classified into categories based on their characteristics [19,20]. Finally, class-specific regressions are applied to derive accurate estimates of PA intensity, sometimes expressed as METs. These estimates are then integrated with time- and user-specific characteristics to predict energy expenditure.

For instance, 2 studies have documented the algorithmic framework used by OMRON (Omron Corporation). At the third processing stage, the OMRON algorithm categorizes accelerometer data into 3 activity types: locomotive, nonlocomotive, or mixed activities [14,20]. However, the locomotive activities considered in its development were limited to walking and running tasks, which involve regular, cyclic alternations of stance and swing phases between the left and right legs.

A Google Scholar search for studies focusing on locomotive behavior recognition using wearable accelerometer sensors highlights a gap in research on skateboard and kickboard activities. This suggests that locomotive activities, characterized by irregular or asymmetric motion patterns, are largely absent in existing algorithms, potentially leading to errors in energy expenditure estimates. Moreover, the unique push-push-glide motion patterns of skateboarding and kickboarding often span several seconds, highlighting the need for longer analytic windows to accurately recognize these behaviors.

### Objectives

While inaccurate feedback from activity trackers or smartphones can discourage users from engaging in active lifestyles [21], this study aims to evaluate the ability of these devices to recognize skateboard and kickboard transportation activities, thereby promoting active commuting habits. The primary objective of this study is to build models that classify sensor data from devices akin to contemporary activity trackers—waist-worn research-grade sensors, wrist-worn consumer-grade devices, and smartphones—by accurately identifying the push-push-glide dynamics of skateboard and kickboard use. The secondary objective is to identify data features that contribute most to the accuracy of the classification models.

## Methods

### Overview of the Study Protocol

A heterogeneous, convenient sample of 10 participants was recruited from the campus of Ochanomizu University and local skate parks to capture a range of skateboarding cruising. Details of participant characteristics are presented in Table 1. Exclusion criteria included being aged <11 years, having any lower limb injury that impaired normal gait, or lacking prior experience with skateboard cruising. Participants followed a structured protocol that involved 9 sedentary and locomotive activities: sitting (while chatting or scrolling a smartphone), standing (while chatting or scrolling a smartphone), bicycling, walking, running, kickboarding, skateboarding, ascending stairs, and descending stairs. Each activity was designed to last at least 10 minutes. This duration was selected to provide enough data samples (approximately 30 nonoverlapping windows of 20 s), while ensuring the entire protocol could be completed within half a day. Some activities required discontinuous data collection due to environment constraints. For instance, stair activities were conducted in an 8-story building, requiring multiple bouts of 2‐3 minutes to achieve a total of 10 minutes. Participants were permitted to stop any activity before reaching the 10-minute mark upon request, to prevent excessive fatigue. All activities were conducted on the campus of Ochanomizu University in Tokyo and were performed in a randomized order to avoid systematic metabolic interferences. Inertial data were collected using wearable sensors, and participants also wore a chest-mounted action camera (Virb 360, Garmin) to record the entire experiment.

**Table 1.** Participant characteristics.

Participant ID	Sex	Age (years)	Skateboarding skill level^a^	Stance^b^	Weight (kg)
001	Woman	12	Expert	Regular	38
002	Woman	26	Beginner	Goofy	42
003	Man	40	Expert	Regular	83
004	Woman	27	Expert	Regular	45
005	Man	22	Beginner	Regular	54
006	Man	22	Expert	Goofy	65
007	Man	13	Expert	Regular	50
008	Man	25	Expert	Goofy	80
009	Man	22	Expert	Regular	65
010	Man	55	Beginner	Regular	65

a Participants categorized as “beginners” reported at least 1 prior experience with skateboarding but were not consistently able to perform the sharp turns required during the experiment.

b Participants with a regular stance naturally position their left foot at the front of the board and kick with their right foot during push-push-glide activities, such as skateboarding. Conversely, participants with a goofy stance naturally place their right foot at the front and kick with their left foot.

### Ethical Considerations

The experimental protocol was approved by the Ochanomizu University research ethics committee (#2021‐10). All participants gave their written consent. To ensure privacy, all data were anonymized before analysis. Some participants did not receive compensation, while others were compensated at an hourly rate ranging from ¥1050 (US $7.10) to ¥1250 (US $8.50), depending on their age and university degree status.

### Data Collection and Preprocessing

**Figure 1.**
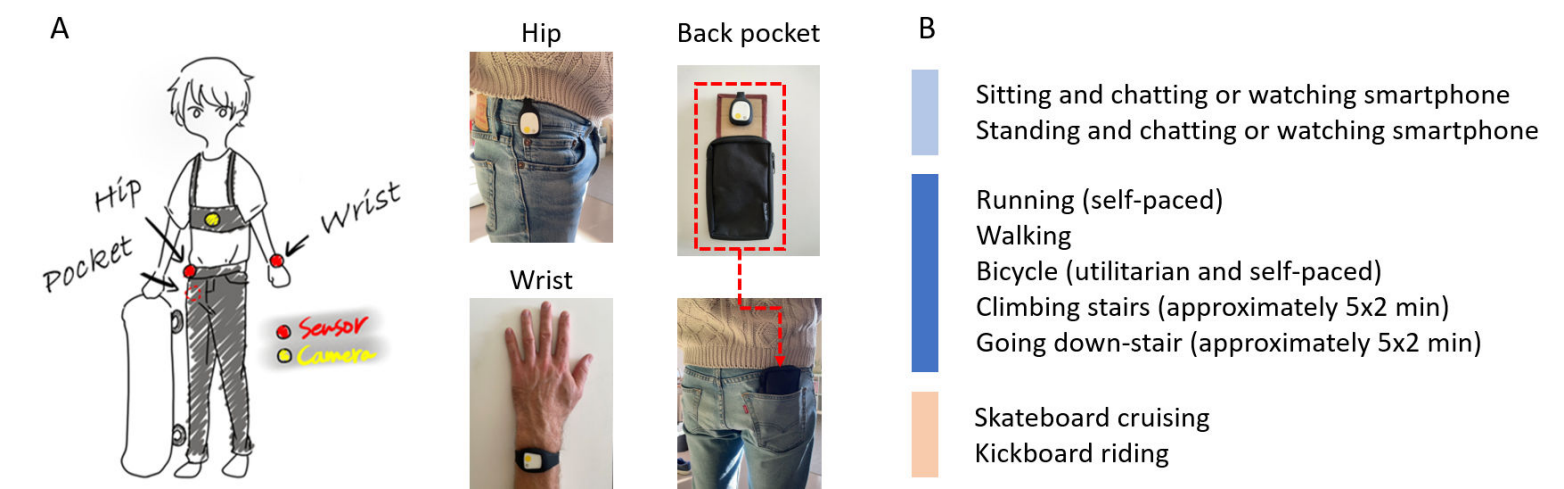
Overview of the materials and protocol. (A) Three MetaMotionS sensors were worn on the left wrist and the right hip and placed in the right back pocket of the trousers. The sensor in the back pocket was clipped onto a piece of cardboard and inserted in a small pouch matching the dimension of a smartphone. An action camera (Virb 360) was mounted on the chest. (B) The participants completed 9 different activities, including 2 sedentary behaviors (light blue); 5 typical locomotive behaviors with symmetrical motion patterns (navy blue); and 2 push-push-glide locomotive activities with asymmetrical motion patterns, that is, skateboard and kickboard cruising (light red). Each activity lasted approximately 10 minutes. Stair activities were performed in an 8-story building and completed in several bouts of approximately 2 minutes each, totaling 10 minutes.

Raw accelerometer, gyroscope, and barometer data were collected using 3 MetaMotionS wearable devices (Mbient Lab) worn on the left wrist and the right hip and placed in the right back pocket of the trousers (Figure 1A). These placements replicated the typical positions for activity trackers and smartphones. The accelerometer and gyroscope sensors were triaxial devices and configured with a sampling rate of 100 Hz, while the barometer sensor sampled at approximately 1 Hz.

The collected data streams were segmented based on the start and end times of each activity. To minimize boundaries effects, the first and last 5 seconds of each activity were removed. The preprocessed data were stored as independent CSV files, resulting in a final dataset comprising 196 files corresponding to the 100 activities performed by the 10 participants. Each file contained 7 independent time series: 6 corresponding to the x-, y-, and z-axis of the accelerometer and gyroscope sensors and 1 corresponding to the barometer sensor. Signal samples for the 9 activities are presented in Multimedia Appendix 1.

### Feature Extraction

Each CSV file was analyzed using sliding analytic windows of 10 or 20 seconds. For each window, 211 data features were extracted from the accelerometer, gyroscope, and barometer data, as follows:

- Accelerometer features*:* 112 features were derived, primarily based on the approach by Willetts et al [16]. These included 29 parameters from the general statistical analysis in the time domain, 12 parameters from the roll-pitch-yaw analysis, and 71 parameters from the fast Fourier transform (FFT) analysis [22,23].
- Gyroscope features*:* 90 features were extracted. These included 30 parameters from general statistical analysis in the time domain and 60 parameters from the FFT analysis.
- Combined features*:* 8 features combining information from the accelerometer and gyroscope sensor were extracted to examine synchrony between the acceleration generated by the push motion and angular velocity during the push-to-glide phases of skateboarding.
- Barometer feature*:* 1 feature was extracted to examine the slope of movements.

Further details on the computation of these features can be found in the data and code repository (refer to “data_preparation.py” for the exact code) [24].

### Configurations

The various combinations of hardware and software used for the analysis replicated the characteristics of various types of commercial devices, as follows:

- Hip sensor*:* analysis of accelerometer data only, reflecting the capabilities of waist-worn research-grade activity trackers [11].
- Pocket sensor: analysis of accelerometer, gyroscope, and barometer data, simulating the capabilities of modern smartphones.
- Wrist sensor (low end): analysis of accelerometer data only, replicating the capabilities of entry-level wrist-worn consumer-grade activity trackers.
- Wrist sensor (high end)*:* analysis of accelerometer, gyroscope, and barometer data simulating the capabilities of high-end, wrist-worn, consumer-grade activity trackers.

### Activity List and Recognition Models

Activity recognition was performed using random forest classifiers across four analyses:

1. Multiclass classification*:* differentiating all 9 activities (shown in Figure 1B).
2. Kickboard versus *rest* and skateboard versus *rest* binary classifications: extracting the kickboard and skateboard estimators trained together using the 1 versus *rest* approach [25] along the full list of activities and applying them to the test samples.
3. Push-push-glide versus *rest* binary classification: grouping kickboard and skateboard activities into a push-push-glide superclass and distinguishing from other activities.
4. Kickboard versus skateboard binary classification: differentiating kickboard and skateboard activities.

Random forests models were configured with 100 trees. For training, data from 8 participants were used, while data from the remaining 2 were reserved for performance evaluation. Due to the data collection protocol, activities had slightly different durations, resulting in small discrepancies in the number of data points available. The training used class balancing to avoid related biases, that is, the weight of each data point during training was normalized by the number of data points available for the corresponding activity. To ensure robustness, the recognition performance was averaged across 100 independent runs for each 8:2 split. The overall performance for each activity list and each sensor configuration (eg, multiclass classification with the pocket sensor) was obtained by averaging the performances across all possible splits. The activity recognition analysis was conducted using *Scikit-learn* [26].

### Feature Importance Analysis

Feature importance quantifies the contribution of each feature. The importance was extracted from the models using the built-in method provided by *Scikit-learn* [26]. This metric is calculated based on the decrease of Gini impurity, which evaluates how effectively each feature separates the different classes [27]. The final importance value for a given feature was calculated by averaging the importance scores obtained across all training repetitions for a specific configuration.

## Results

### Participant Characteristics

As shown in Table 1, the group of participants included 4 women and 6 men, aged between 12 and 55 (mean 26, SD 12) years, with weights ranging from 38 to 83 (mean 59, SD 15) kg. Seven participants were comfortable riding a skateboard, while 3 were classified as beginners, as they were unable to perform sharp turns and occasionally needed to step off and manually adjust the direction of the board. All participants were comfortable using a kickboard.

### Multiclass Classification Models

**Figure 2.**
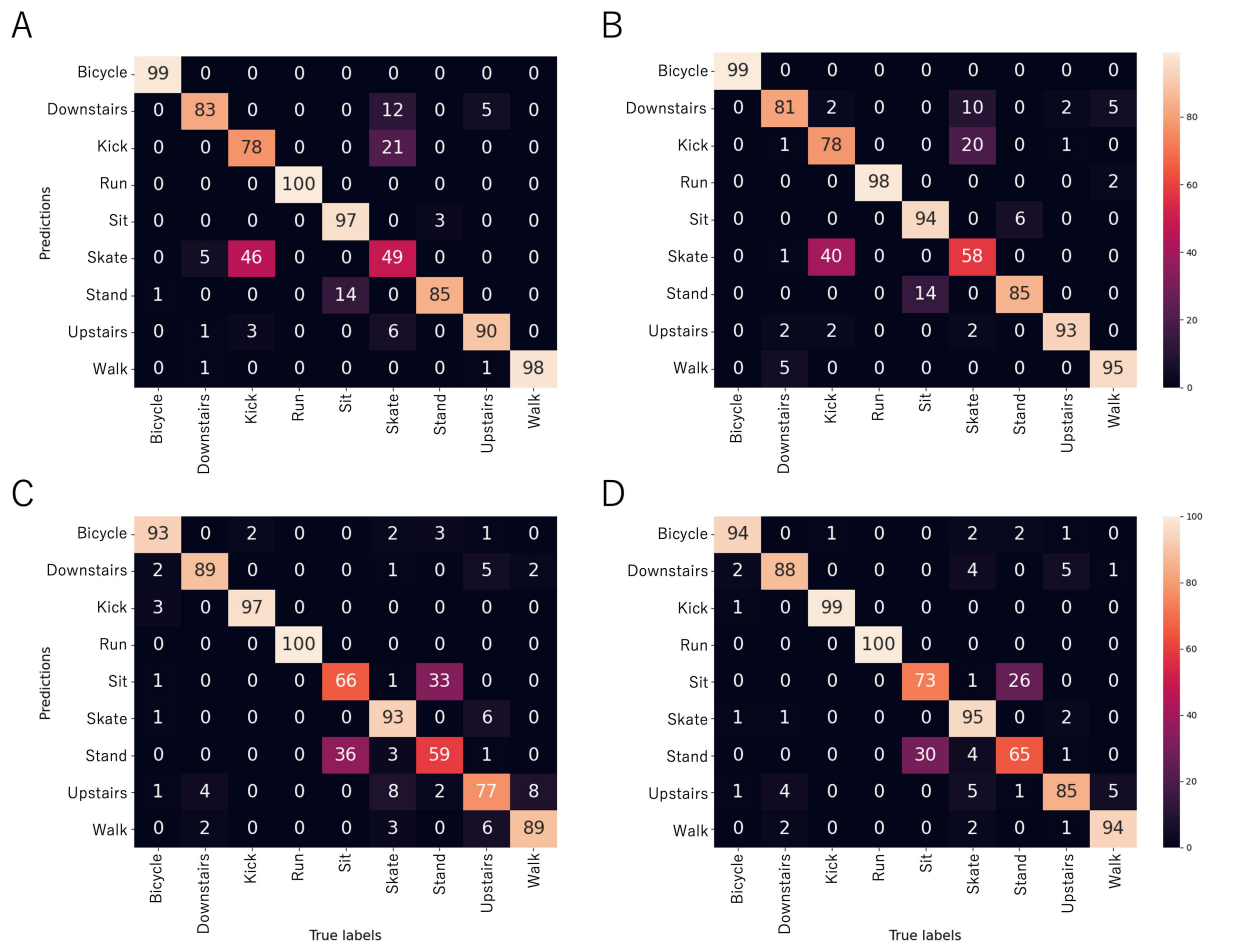
Confusion matrices for each device and configuration and for the multiactivity classification models (20-s window results). For each activity, values refer to the normalized sensitivity. (**A**) Hip sensor and accelerometer-derived features; replicates the configuration of waist-worn research-grade activity trackers. (**B**) Pocket sensor and accelerometer-, gyroscope-, and barometer-derived features; replicates the configuration of contemporary smartphone devices. (**C**) Wrist sensor and accelerometer-derived features; replicates the configuration of entry-level wrist-worn activity trackers. (**D**) Wrist sensor and accelerometer-, gyroscope-, and barometer-derived features; replicates the configuration of high-end, wrist-worn activity trackers. “Downstairs” denotes descending stairs, “Kick” denotes kickboard, “Skate” denotes skateboard, and “Upstairs” denotes ascending stairs.

As shown in Table 2, the balanced accuracies for the multiclass classification models ranged from 84% (SD 3%; wrist sensor using accelerometer data with 10-s analytic windows) to 88% (SD 3%; wrist sensor using accelerometer, gyroscope, and barometer data with 10- or 20-s analytic windows). The size of the analytic window did not affect the overall performance of the models. The confusion matrices presented in Figure 2 highlight lower performance of models using hip sensor accelerometer data or the pocket sensor accelerometer, gyroscope, and barometer data for recognizing skateboarding and kickboarding. Skateboarding was correctly classified in only 49% (SD 23%; across train-test splits) of cases by the hip sensor models (Figure 2A) and in 58% (SD 32%; across train-test splits) of cases by the pocket sensor models (Figure 2B). Kickboarding was accurately classified in 78% (SD 20%) of cases by the hip sensor and the pocket sensor models. Most classification errors involving these 2 activities were due to mutual confusion. Figure 2 shows the results for the 20-second window analysis only. The 10-second window analysis resulted in similar scores, which are presented in Multimedia Appendix 1.

**Table 2.** Balance accuracies for each device, configuration, and classification model (10-s and 20-s window results).

Classification task		Hip sensor and features from accelerometer data^a^ (%), mean (SD)	Pocket sensor and features from accelerometer, gyroscope, and barometer data^b^ (%), mean (SD)	Wrist sensor and features from accelerometer data^c^ (%), mean (SD)	Wrist sensor and features from accelerometer, gyroscope, and barometer data^d^ (%), mean (SD)
Multiclass					
	10-s window	86 (5)	86 (5)	84 (3)	88 (3)
	20-s window	87 (5)	87 (5)	85 (4)	88 (3)
Kickboard versus *rest*					
	10-s window	70 (9)	71 (13)	89 (10)	91 (8)
	20-s window	71 (9)	71 (13)	87 (10)	90 (9)
Skateboard versus *rest*					
	10-s window	56 (7)	65 (12)	84 (9)	83 (11)
	20-s window	55 (7)	67 (13)	83 (10)	83 (11)
Push-push-glide versus *rest*					
	10-s window	92 (6)	95 (3)	91 (7)	91 (8)
	20-s window	93 (7)	95 (4)	91 (6)	92 (8)
Kickboard versus skateboard					
	10-s window	65 (12)	65 (18)	100 (1)	100 (0)
	20-s window	66 (12)	68 (15)	100 (0)	100 (0)

a The configuration of the hip sensor and accelerometer-derived features replicates that of waist-worn research-grade activity trackers.

b The configuration of the pocket sensor and accelerometer-, gyroscope-, and barometer-derived features replicates that of contemporary smartphone devices.

c The configuration of the wrist sensor and accelerometer-derived features replicates that of entry-level wrist-worn activity trackers.

d The configuration of the wrist sensor and accelerometer-, gyroscope-, and barometer-derived features replicates that of high-end, wrist-worn activity trackers.

### Binary Classification Models

**Figure 3.**
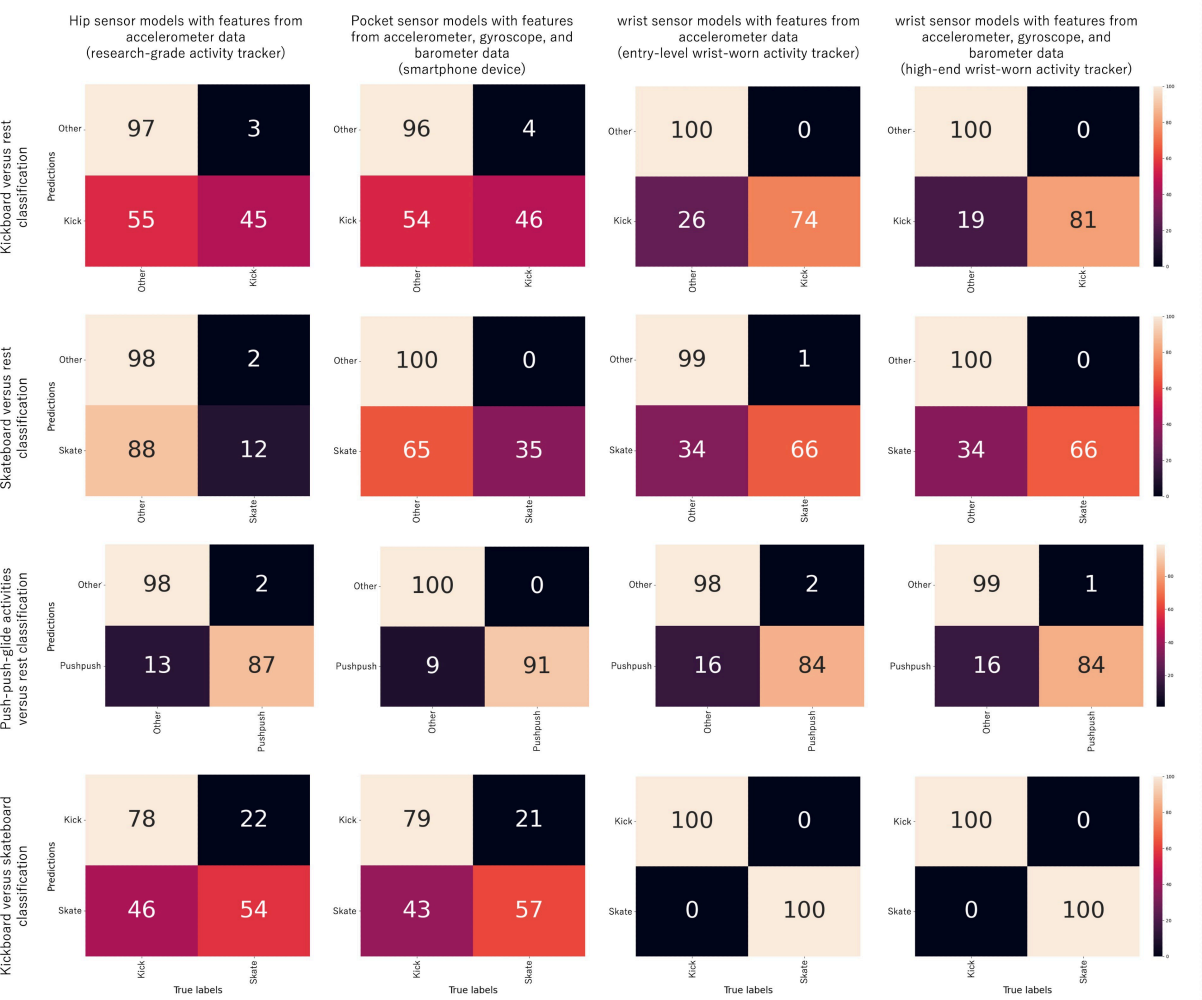
Confusion matrices for each device, configuration, and binary classification model (20-s window results). For each activity, values refer to the normalized sensitivity. “Kick” denotes kickboard, “Pushpush” denotes push-push-glide activities, and “Skate” denotes skateboard.

Detailed results are shown in Figure 3. The balanced accuracies for the kickboard versus *rest* classification ranged from 70% (SD 9%) to 91% (SD 8%). Higher accuracies were observed for the wrist sensor models, regardless of whether only accelerometer data or a combination of accelerometer, gyroscope, and barometer data was used. The skateboard versus *rest* classification models demonstrated lower balanced accuracies, ranging from 55% (SD 7%) to 84% (SD 9%). Similar to kickboard versus *rest*, higher accuracies were observed for the wrist sensor models.

The push-push-glide versus *rest* classification showed consistently high balanced accuracies, ranging from 91% (SD 8%) to 95% (SD 4%). In contrast, the kickboard versus skateboard classification models achieved balanced accuracies ranging from 65% (SD 12%; pocket sensor using accelerometer, gyroscope, and barometer data with 10 s analytic windows) to 100% (SD 0%; wrist sensor using accelerometer, gyroscope, and barometer data and with 10-s analytic windows or the wrist sensor with any configuration using the 20-s analytic windows).

All balanced accuracy scores are presented in Table 2. The performances remained consistent across models, regardless of whether 10- and 20-second analytic windows were used.

### Feature Importance

A list of the 10 most important features used in the binary classifications is shown in Multimedia Appendix 2. A large number of the most common features are shared across configurations and classification targets (kickboard vs *rest*, skateboard vs *rest*, both vs *rest*, and kickboard vs skateboard). This is particularly the case for statistics features based on the norm of the acceleration vector, such as the skewness (9 uses), kurtosis (7 uses), or its FFT, in particular low frequencies (8 uses for 1‐2 Hz and 7 uses for 0‐1 Hz) and ratio of maximum power to total power (6 uses). In addition, the pitch and yaw of the acceleration vector relative to the gravity vector are among the most used features (7 and 6 uses, respectively). Beside the norm of the acceleration vector, the average of its y-axis and z-axis components is also strongly represented (6 uses each).

The remaining features tend to have a low use count but similar interpretations. For wrist sensor configurations, we have a large number of FFT features related to high frequencies (10‐15 Hz and 20 combined uses).

## Discussion

### Main Findings

In recent years, kickboarding and skateboarding have become increasingly common modes of transportation [5-7]. However, there is a lack of algorithms capable of accurately recognizing these behaviors, which impacts the accuracy of daily PA estimates from activity trackers. Such inconsistencies may discourage users from engaging in active transportation behaviors [21].

In this study, accelerometer data—alone or combined with gyroscope and barometer data—collected from devices mimicking contemporary activity trackers or smartphone sensing capabilities were used to develop classification models capable of recognizing kickboarding and skateboarding commuting behaviors. A total of 112 accelerometer-derived features were extracted, as described in previous studies [22,23]. In addition, 99 features based on gyroscope and barometer data were extracted, some of which were specifically designed to capture the irregular and asymmetric motion patterns of kickboarding or skateboarding. These features were expected to capture the unique characteristics of the push-push-glide motion. Feature extraction was conducted using 10- and 20-second sliding window analyses. Random forest algorithms using subject-wise training-test assignments were used to develop multiclass and binary classification activity recognition models across four sensor configurations: (1) hip sensor with accelerometer data only; (2) pocket sensor with the accelerometer, gyroscope, and barometer data; (3) wrist sensor with accelerometer data only; and (4) wrist sensor with the accelerometer, gyroscope, and barometer data. The wrist sensor multiclass classification models achieved high sensitivities for recognizing kickboarding (range 97%‐99%) and skateboarding (range 93%‐95%). In contrast, the hip and pocket sensor multiclass models exhibited lower performance for these activities, with sensitivities of 78% for kickboarding and 49% to 58% for skateboarding. Most classification errors emanated from confusion between kickboarding and skateboarding. In addition, the low number of participants with a goofy stance may have affected model training, as their movement patterns are inherently opposite to those of regular riders. Future studies would benefit from a higher number of goofy-stance participants to improve the generalizability of the results. Binary classification models distinguishing push-push-glide activities from others significantly improve recognition accuracy.

### Kickboard and Skateboard Recognition by the Hip and Pocket Sensor Configurations

The multiclass classification models achieved balanced accuracies ranging from 84% to 87%, comparable to those obtained with the hip and pocket sensor configurations (Table 2). The confusion matrices shown in Figures 2A and 2B indicated high accuracy of the estimators for the 7 activities unrelated to kickboarding or skateboarding. These findings are consistent with those of Suker et al [28], who reported classification rates of approximately 90% for recognizing sedentary and locomotive activities (excluding kickboarding and skateboarding) using accelerometer data from a smartphone sensor placed in the pocket. The recognition of kickboarding and skateboarding by the multiclass classification models proved more challenging. In particular, skateboarding yielded sensitivities of 49% with the hip sensor using accelerometer data and 58% with the pocket sensor using the accelerometer, gyroscope, and barometer data. Most classification errors arose from confusions between these 2 activities. This observation led to the testing of binary classification models considering a push-push-glide activity class versus all other activities. These models achieved balanced accuracies ranging from 92% to 95%. However, binary models distinguishing between kickboarding and skateboarding did not outperform the multiclass classification models, discouraging the implementation of a multilevel algorithmic procedure involving the initial recognition of push-push-glide activities followed by differentiation between kickboarding and skateboarding. Given the motion similarities between the 2 activities in the context of commuting, software applications designed to evaluate energy expenditure for these activities might consider halting the classification process at the push-push-glide activity recognition stage. Further studies investigating the energy expenditure associated with kickboarding and skateboarding in commuting contexts are necessary to validate this approach.

### Kickboard and Skateboard Recognition by the Wrist Sensor Configurations

The performances of the multiclass classification models were promising, with balanced accuracies of 84% for wrist sensor with accelerometer data only and 88% for the wrist sensor with the accelerometer, gyroscope, and barometer data (Table 2). Interestingly, all locomotive activities including skateboarding and kickboarding achieved sensitivity scores of approximately ≥90%, except for the ascending stairs activity, which exhibited confusions with various activities (Figures 2C and 2D). Such values are on par with or above performances found in the literature for random forest models [29]. Confusions among sedentary activities, such as sitting or standing while chatting or scrolling a smartphone, also contributed to lower balanced accuracies in the multiclass classification models. These results should not overshadow the main objectives of this study. First, the primary aim was to evaluate the feasibility of recognizing kickboarding and skateboarding commuting behaviors. Second, distinguishing between sedentary and locomotive behaviors has been a standard feature in activity tracker software for over a decade [14,20]. It may be suggested that specific methods be developed to further improve the recognition of sitting and standing, but this was beyond the scope of this study.

With regard to the primary aim, kickboarding and skateboarding achieved sensitivity scores of ≥93% in the multiclass models. Binary classification models (eg, kickboard vs *rest*, skateboard vs *rest*, push-push-glide activities vs *rest*, and kickboard vs skateboard) were tested to improve performance. However, in all cases, binary models performed worse than the multiclass models. This discrepancy may stem from the lack of concurrent classification processes in binary models. For instance, even if a data point is moderately likely to belong to one activity class (eg, kickboarding), it might be far more likely to belong to another activity class (eg, skateboarding), which is captured more effectively in multiclass models. In those scenarios, eliminating 1 activity during evaluation yielded improved results (Multimedia Appendix 1), consistent with findings from models classifying push-push-glide activities versus all other activities. While binary classification models distinguishing kickboarding from skateboarding achieved 100% accuracies, confusion matrices for the push-push-glide activities versus *rest* revealed that 16% (SD 0.2%) of kickboarding or skateboarding activities were misclassified.

This suggests that a multilevel classification model, beginning with the recognition of push-push-glide activities and followed by differentiation between kickboarding and skateboarding, is unlikely to outperform direct multiclass classification models. Further studies conducted in less controlled environments will be necessary to identify the optimal approach for recognizing kickboarding and skateboarding behaviors.

### Features

The analysis of feature importance enhances model explainability by providing insights into the classification process. First, data shown in Multimedia Appendix 2 highlight features extracted from roll-pitch-yaw analysis as recurrent variables in the classification models for the hip and pocket sensor configurations. These features likely capture the leg extension during the push movement, which is more pronounced in kickboarding and skateboarding compared to other locomotive activities such as walking, running, and cycling. Features capturing low-frequency information are also highly represented, as the glide phase has a long duration, and the push (or propulsion) phase is extended compared to walking or running. Regarding wrist sensor configurations, FFT feature analysis revealed a prominence of signals in higher-frequency bands (ie, >10 Hz), likely due to vibrations from ground surface imperfections. Activity tracker software designed to recognize kickboarding and skateboarding may need to analyze signals in these higher frequencies—an approach that may not currently be standard, given that human movement typically occurs at lower-frequency ranges. Second, the analysis of the top features used to classify kickboard versus skateboard helps explain the difficulty in distinguishing these activities in the kickboard versus *rest* and skateboard versus *rest* models. Only the wrist sensor configurations could strongly differentiate the 2 activities. The feature named “accelerometer, z-axis, mean” captures the fact that the hands remain on the handlebar while kickboarding, whereas yaw measurement provides information about the wrist orientation, further aiding the differentiation. Third, the distribution of feature values across kickboarding, skateboarding, or other activities (Multimedia Appendix 1) revealed distinct activity-based patterns but also substantial overlaps. These overlaps cover a broad region of the feature space, supporting the hypothesis that treating *rest* activities as a single category increases the classification difficulty.

Beyond improving model interpretability, the analysis of feature importance has practical implications for industrial applications, particularly in optimizing memory and computational efficiency. For instance, in the push-push-glide versus *rest* classification task, the wrist sensor configuration using gyroscope data relied on a different set of accelerometer-based features compared to the wrist sensor configuration using accelerometer data only. Given that both configurations achieved comparable classification performance, this suggests redundancy in the extracted information, as expected. Consequently, the number of features could likely be reduced without compromising classification accuracy, thereby streamlining computational or hardware requirements for integration into commercial activity trackers.

This study used features previously suggested in the literature for recognizing daily life activities [16,22,23]. Such studies usually focus on common locomotive activities such as walking, running, and cycling. To address the challenge of recognizing kickboarding and skateboarding behaviors, a set of features was designed to capture the specific motion frequencies of these activities, as well as the body rotation specific to skateboarding. However, additional research may be needed to develop further features tailored to these activities.

### Conclusions

This study demonstrates the feasibility of developing activity tracker software capable of classifying commuting behaviors such as kickboarding and skateboarding. Despite a relatively small sample size of 10 participants, the heterogeneity of the population (age, sex, riding skills, and stance) provided enough information for the machine learning algorithm to perform well, validating the approach. The wrist sensor configuration showed promising results, achieving high accuracy across locomotive activities, including kickboarding and skateboarding. In contrast, classification performances for hip and pocket sensor configurations were more variable, particularly in distinguishing between kickboarding and skateboarding, likely due to the similar lower limb motion patterns involved. Nevertheless, the recognition of the broader push-push-glide activity category achieved acceptable accuracies (range 87%‐91%), suggesting its potential as a practical intermediate classification step. Accurate classification would enable activity tracker software to implement intraclass regression algorithms for more precise predictions of energy expenditure associated with kickboarding and skateboarding commuting behaviors. However, future studies using a larger dataset are required to train models that could be deployed in existing commercial activity trackers. Finally, considering the respective strengths of wrist sensor and hip and pocket sensor configurations, models using sensor fusion approaches could further enhance classification performance.

## Supplementary material

10.2196/71969 Multimedia Appendix 1 Additional results.

10.2196/71969 Multimedia Appendix 2 Identification of the most important features.

## Abbreviations

FFT: fast Fourier transform
MET: metabolic equivalent of task
PA: physical activity
