# Supplementary material for: Recognizing Skateboard and Kickboard Commuting Behaviors Using Activity Trackers: Feasibility Study Using Machine Learning Approaches
Source: JMIR Form Res. 2025 Aug 29;9:e71969. doi: 10.2196/71969 (PMC12396795; doi:10.2196/71969)
Supplement: Multimedia Appendix 1 [file formative-v9-e71969-s001.pdf]

Multimedia appendix 1: additional results.

1. *Confusion matrices for the multi-class analysis using 10-second analytic windows [page 2]*
2. *Confusion matrices for the kickboard vs. rest analysis using 10-second analytic windows [page 4]*
3. *Confusion matrices for the skateboard vs. rest analysis using 10-second analytic windows [page 6]*
4. *Confusion matrices for the push-push-glide vs. rest analysis using 10-second analytic windows [page 8]*
5. *Confusion matrices for the kickboard vs. skateboard analysis using 10-second analytic windows [page 10]*
6. *Feature importance for the hip sensor (accelerometer configuration) [page 12]*
7. *Feature importance for the pocket sensor (accelerometer, gyroscope, barometer) [page 12]*
8. *Feature importance for the wrist sensor (accelerometer configuration) [page 13]*
9. *Feature importance for the wrist sensor (accelerometer, gyroscope, barometer) [page 13]*
10. *Codebook for the importance feature [page 14]*
11. *Visualization for some selected features [page16]*
12. *Confusion matrices for the kickboard vs. rest analysis removing skateboard data (20-second windows) [page 18]*
13. *Confusion matrices for the skateboard vs. rest analysis removing kickboard data (20-second windows) [page 20]*

1. Confusion matrices for the multi-class analysis using 10-second analytic windows

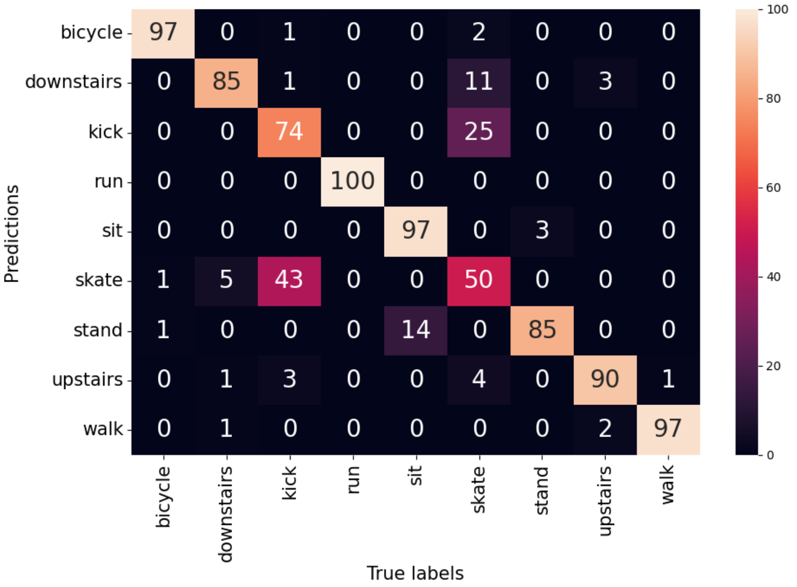

Hip sensor and accelerometer-derived features (waist-worn research-grade activity tracker configuration)

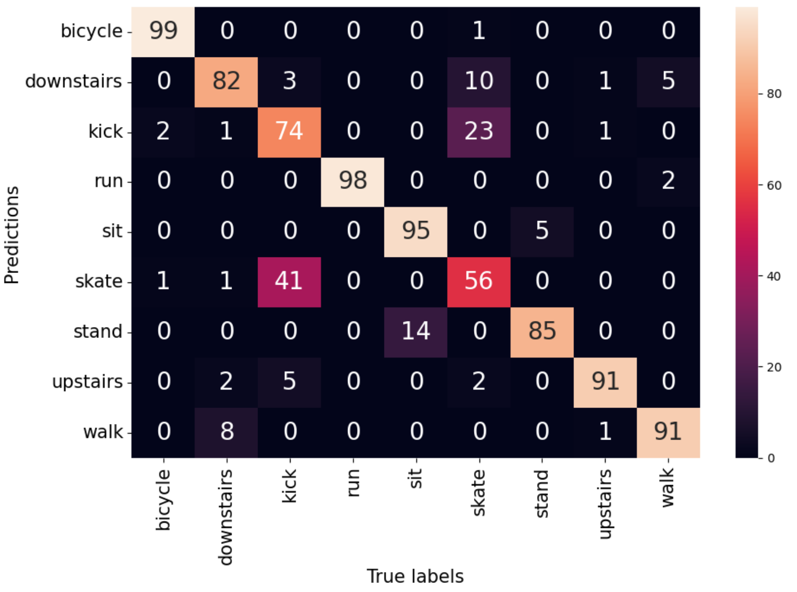

Pocket sensor and accelerometer-, gyroscope-, and barometer-derived features (contemporary smartphone device configuration)

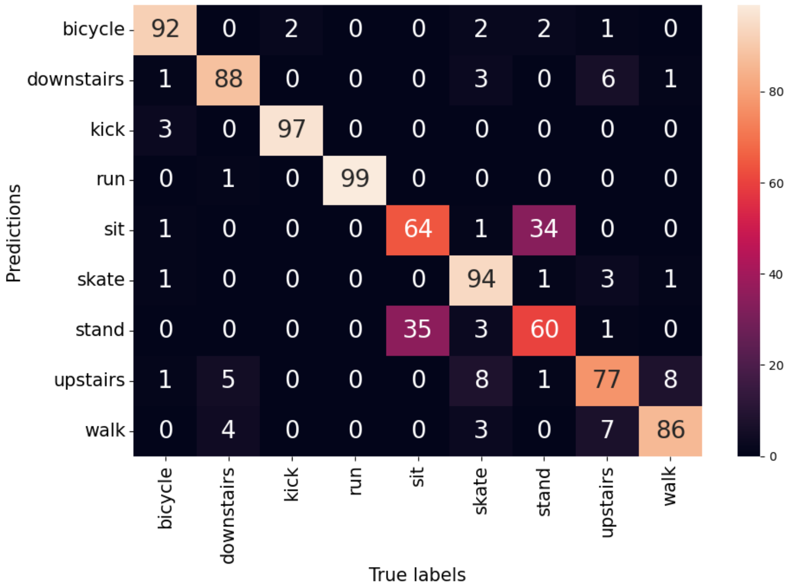

Wrist sensor and accelerometer-derived features
(entry level wrist-worn activity tracker configuration)

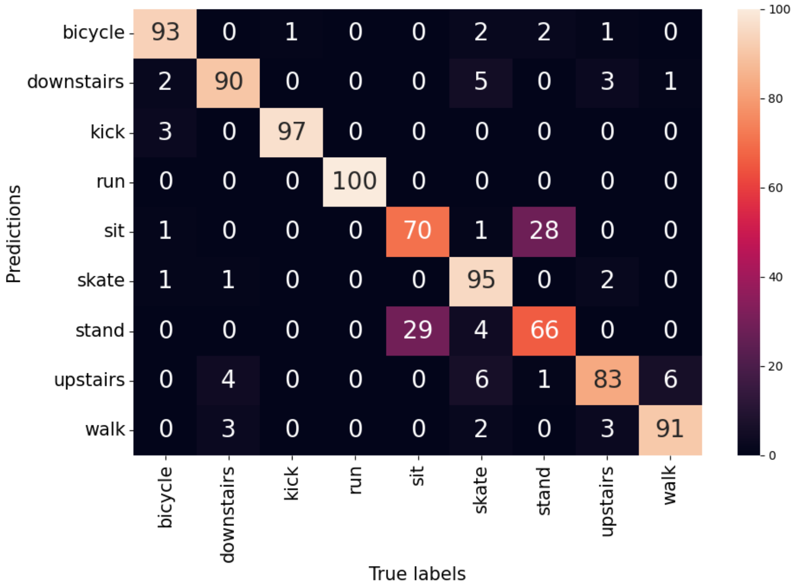

Wrist sensor and accelerometer-, gyroscope-, and barometer-derived features
(high-end wrist-worn activity tracker configuration)

2. *Confusion matrices for the kickboard vs. rest analysis using 10-second analytic windows*

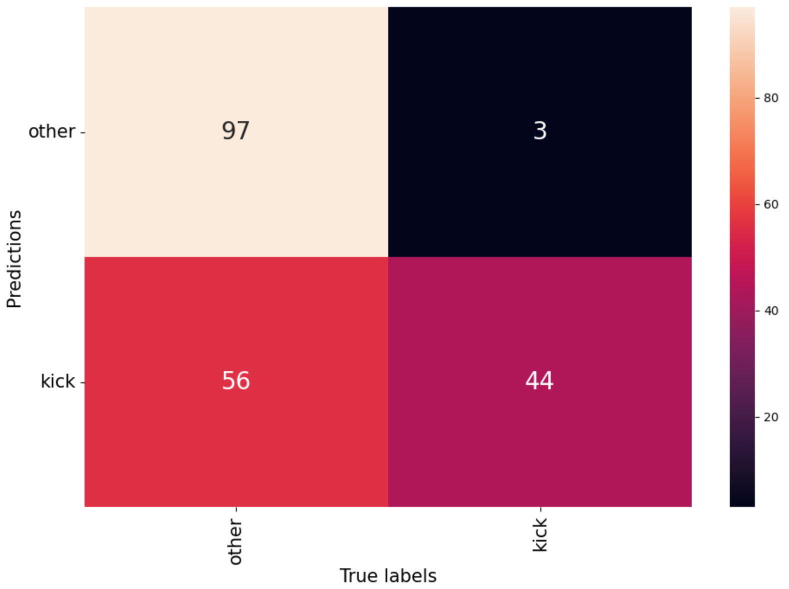

Hip sensor and accelerometer-derived features (waist-worn research-grade activity tracker configuration)

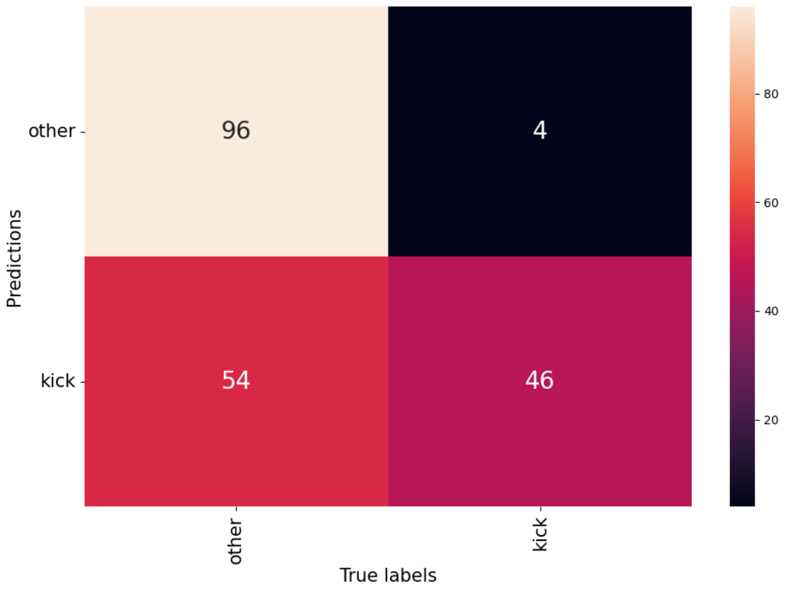

Pocket sensor and accelerometer-, gyroscope-, and barometer-derived features (contemporary smartphone device configuration)

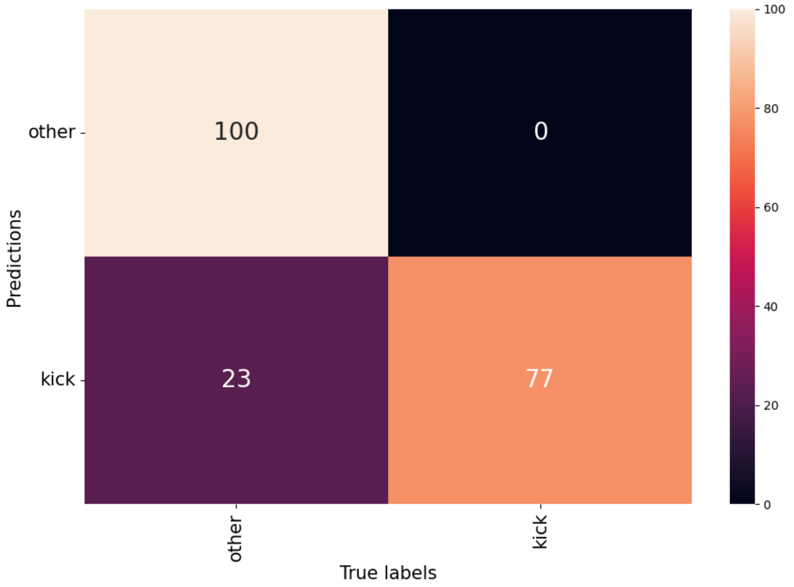

Wrist sensor and accelerometer-derived features
(entry level wrist-worn activity tracker configuration)

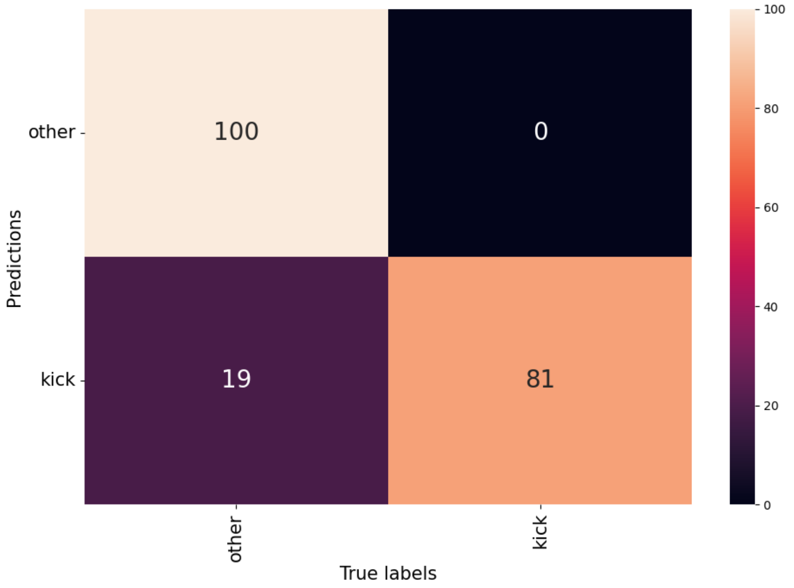

Wrist sensor and accelerometer-, gyroscope-, and barometer-derived features
(high-end wrist-worn activity tracker configuration)

3. *Confusion matrices for the skateboard vs. rest analysis using 10-second analytic windows*

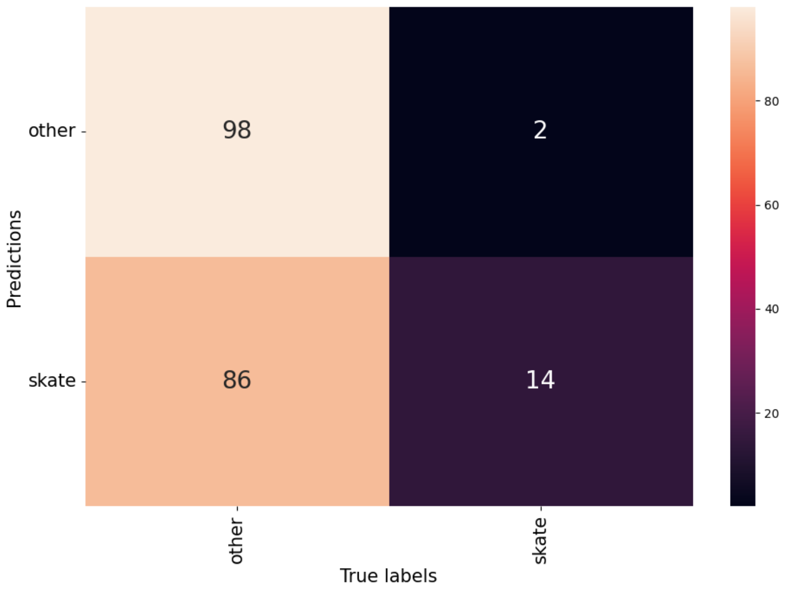

Hip sensor and accelerometer-derived features
(waist-worn research-grade activity tracker configuration)

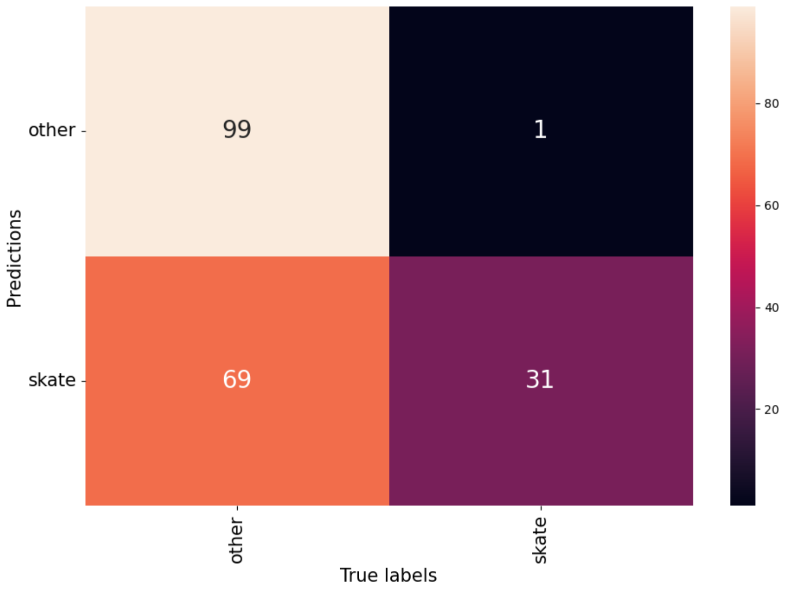

Pocket sensor and accelerometer-, gyroscope-, and barometer-derived features
(contemporary smartphone device configuration)

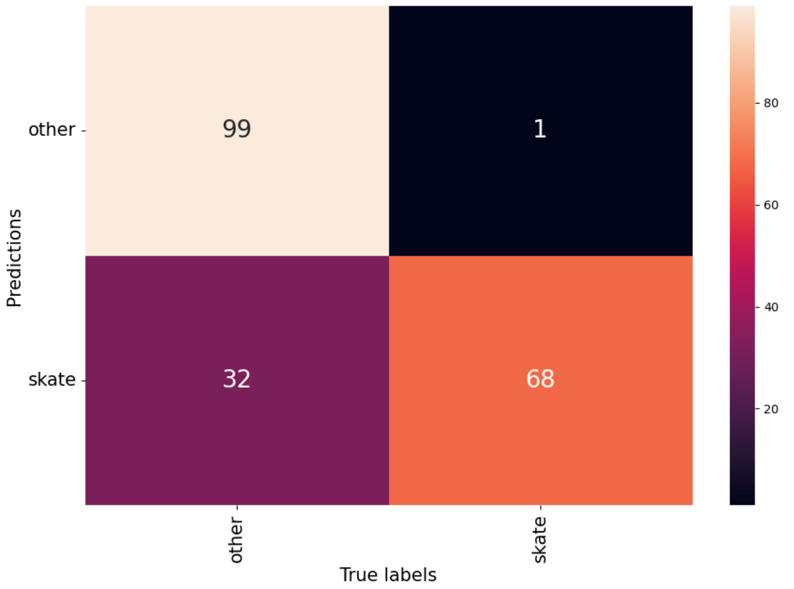

Wrist sensor and accelerometer-derived features (entry level wrist-worn activity tracker configuration)

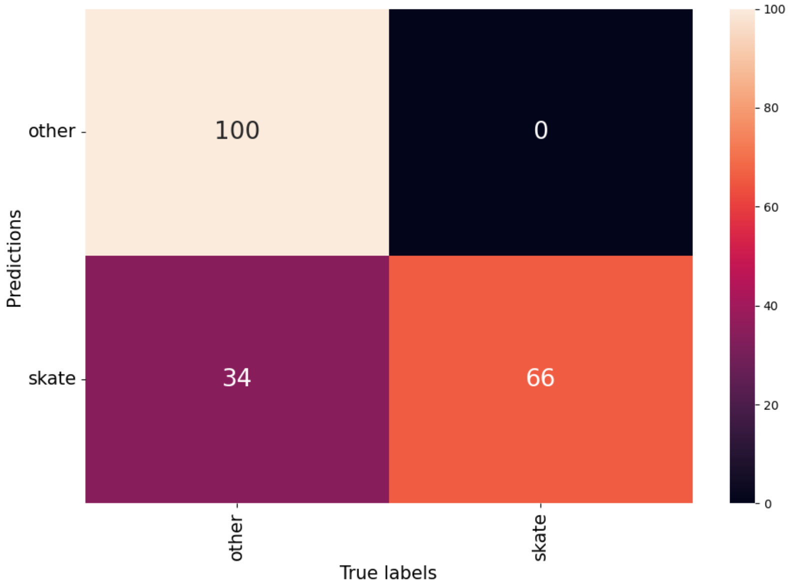

Wrist sensor and accelerometer-, gyroscope-, and barometer-derived features (high-end wrist-worn activity tracker configuration)

4. *Confusion matrices for the push-push-glide vs. rest analysis using 10-second analytic windows*

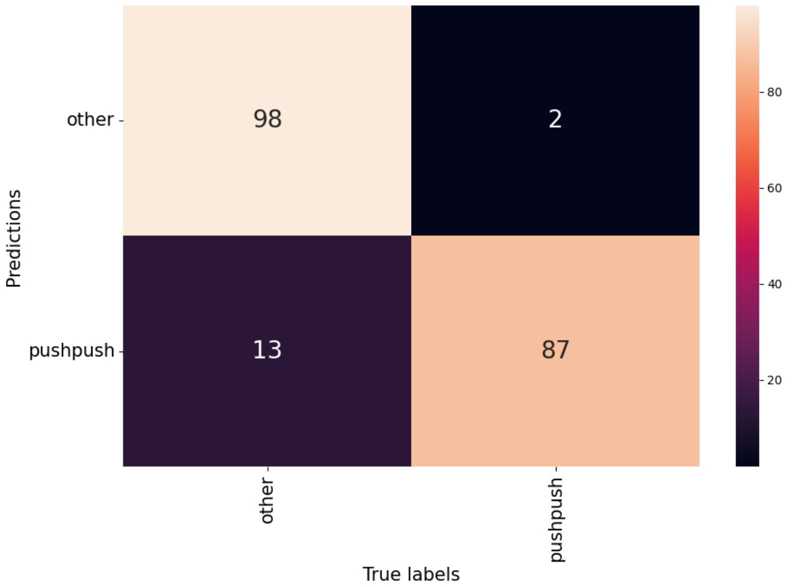

Hip sensor and accelerometer-derived features (waist-worn research-grade activity tracker configuration)

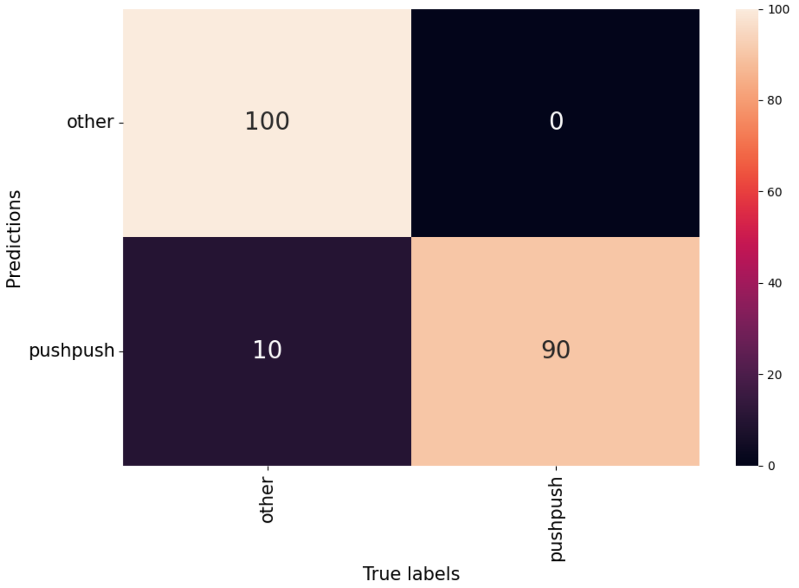

Pocket sensor and accelerometer-, gyroscope-, and barometer-derived features (contemporary smartphone device configuration)

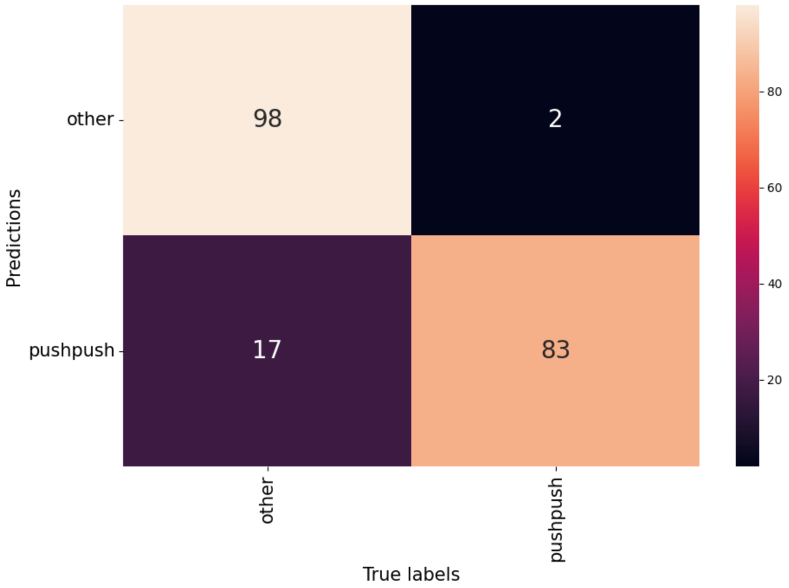

Wrist sensor and accelerometer-derived features
(entry level wrist-worn activity tracker configuration)

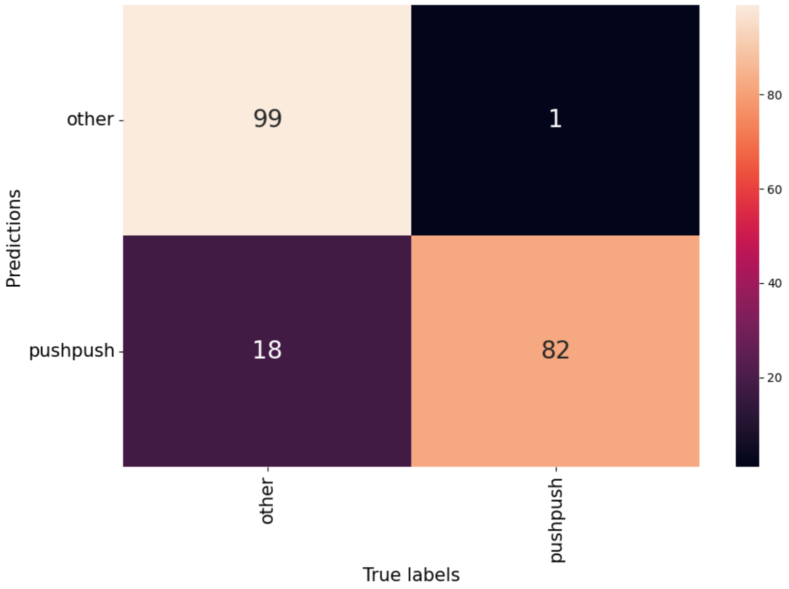

Wrist sensor and accelerometer-, gyroscope-, and barometer-derived features
(high-end wrist-worn activity tracker configuration)

5. Confusion matrices for the kickboard vs. skateboard analysis using 10-second analytic windows

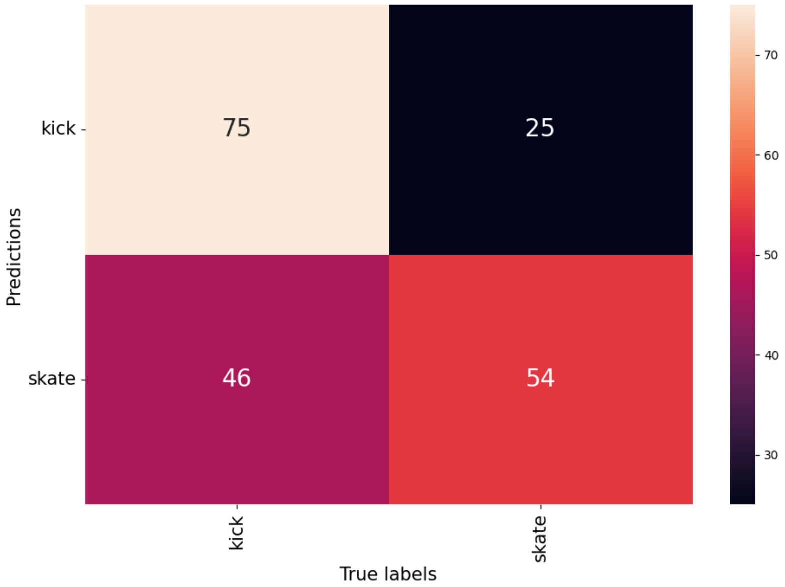

Hip sensor and accelerometer-derived features
(waist-worn research-grade activity tracker configuration)

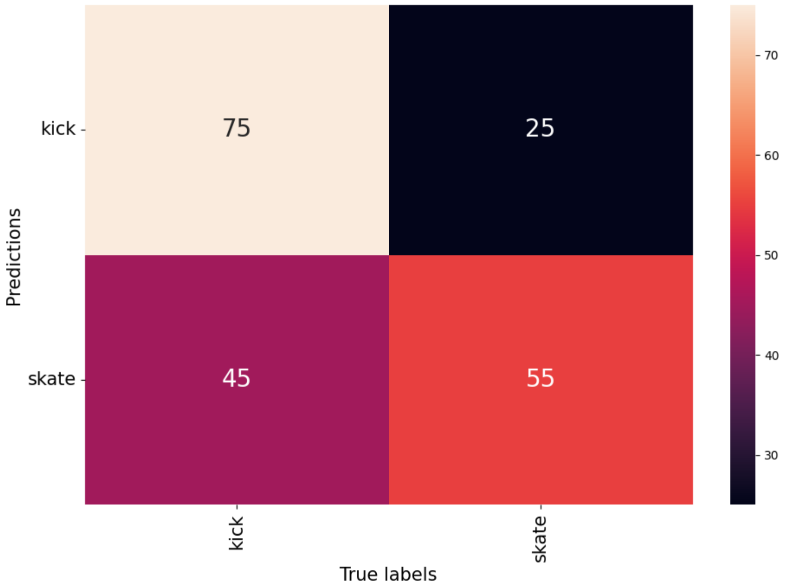

Pocket sensor and accelerometer-, gyroscope-, and barometer-derived features
(contemporary smartphone device configuration)

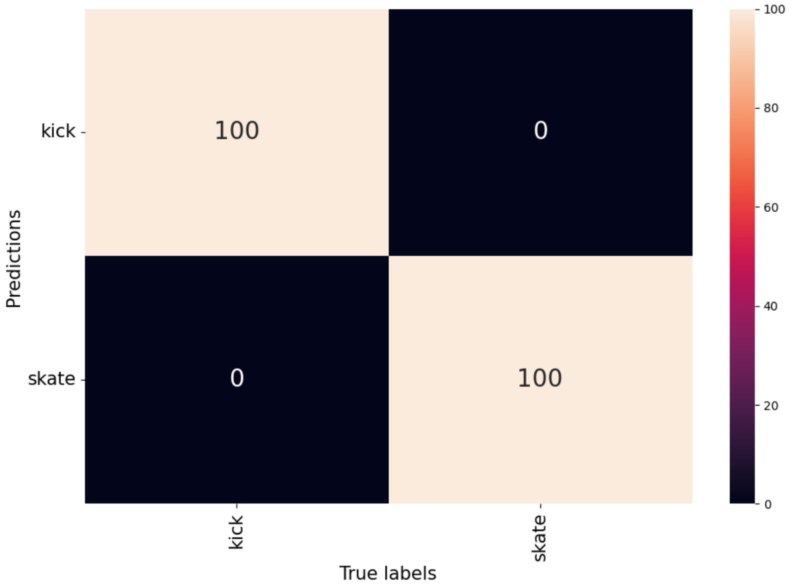

Wrist sensor and accelerometer-derived features
(entry level wrist-worn activity tracker configuration)

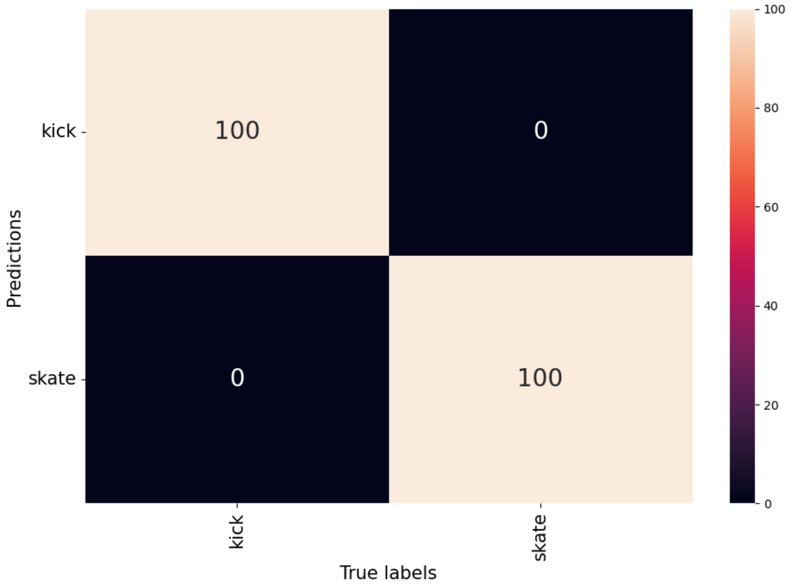

Wrist sensor and accelerometer-, gyroscope-, and barometer-derived features
(high-end wrist-worn activity tracker configuration)

6. *Feature importance for the hip sensor (accelerometer configuration)*

Importance Rank	1	2	3	4	5	6	7	8	9	10
Kickboard vs. rest	Accelerometer, y-axis, FFT, 0–1 Hz band	Accelerometer, vector norm, FFT, 0–1 Hz band	Accelerometer, mean yaw	Accelerometer, roll standard deviation, relative to gravity direction	Accelerometer, mean roll	Accelerometer, pitch standard deviation, relative to gravity direction	Accelerometer, vector norm, skewness	Accelerometer, mean pitch, relative to gravity direction	Accelerometer, y-axis, mean	Accelerometer, x-axis, FFT, 0–1 Hz band
Skateboard vs. rest	Accelerometer, vector norm, FFT, 0–1 Hz band	Accelerometer, vector norm, skewness	Accelerometer, roll standard deviation, relative to gravity direction	Accelerometer, pitch standard deviation, relative to gravity direction	Accelerometer, vector norm, FFT, 1–2 Hz band	Accelerometer, vector norm, FFT, ratio maximum power on total power	Accelerometer, y-axis, FFT, 0–1 Hz band	Accelerometer, vector norm, median	Accelerometer, x-axis, FFT, 1–2 Hz band	Accelerometer, kurtosis
Push-push-glide vs. rest	Accelerometer, vector norm, FFT, 0–1 Hz band	Accelerometer, vector norm, skewness	Accelerometer, y-axis, FFT, 0–1 Hz band	Accelerometer, pitch standard deviation, relative to gravity direction	Accelerometer, vector norm, FFT, ratio maximum power on total power	Accelerometer, roll standard deviation, relative to gravity direction	Accelerometer, kurtosis	Accelerometer, x-axis, FFT, 1–2 Hz band	Accelerometer, vector norm, FFT, 1–2 Hz band	Accelerometer, x-axis, FFT, 0–1 Hz band
Kickboard vs. skateboard	Accelerometer, vector norm, median	Accelerometer, mean yaw	Accelerometer, y-axis, mean	Accelerometer, yaw standard deviation	Accelerometer, mean roll	Accelerometer, mean pitch, relative to gravity direction	Accelerometer, vector norm, FFT, 1–2 Hz band	Accelerometer, y-axis, FFT, 5–6 Hz band	Accelerometer, z-axis, FFT, 7–8 Hz band	Accelerometer, roll standard deviation, relative to gravity direction

4
3
2
1

Identification of the most important features for the hip sensor (accelerometer configuration) only.

7. *Feature importance for the pocket sensor (accelerometer, gyroscope, barometer)*

Importance rank	1	2	3	4	5	6	7	8	9	10
Kickboard vs. rest	Accelerometer, vector norm, FFT, 0–1 Hz band	Accelerometer, vector norm, skewness	Gyroscope, vector norm, FFT, 0–1 Hz band	Accelerometer, yaw standard deviation, relative to gravity direction	Accelerometer, vector norm, FFT, ratio maximum power on total power	Accelerometer, vector norm, FFT, 1–2 Hz band	Accelerometer, kurtosis	Accelerometer, z-axis, FFT, 0–1 Hz band	Gyroscope, z-axis, 25th percentile	Accelerometer, pitch standard deviation, relative to gravity direction
Skateboard vs. rest	Accelerometer, vector norm, gyroscope, y-axis, FFT, correlation	Accelerometer, vector norm, gyroscope, z-axis, FFT, correlation	Accelerometer, vector norm, FFT, 0–1 Hz band	Accelerometer, vector norm, x-axis, FFT, correlation	Accelerometer, vector norm, gyroscope, x-axis, FFT, correlation	Accelerometer, vector norm, FFT, ratio maximum power on total power	Accelerometer, z-axis, FFT, 0–1 Hz band	Accelerometer, z-axis, FFT, 1–2 Hz band	Accelerometer, vector norm, skewness	Accelerometer, mean pitch, relative to gravity direction
Push-push-glide vs. rest	Accelerometer, vector norm, skewness	Accelerometer, vector norm, FFT, 0–1 Hz band	Accelerometer, vector norm, gyroscope, y-axis, FFT, correlation	Accelerometer, vector norm, FFT, ratio maximum power on total power	Accelerometer, vector norm, gyroscope, x-axis, FFT, correlation	Accelerometer, z-axis, FFT, 0–1 Hz band	Accelerometer, kurtosis	Gyroscope, vector norm, FFT, 0–1 Hz band	Accelerometer, vector norm, gyroscope, z-axis, FFT, correlation	Accelerometer, yaw standard deviation, relative to gravity direction
Kickboard vs. skateboard	Accelerometer, mean roll	Accelerometer, mean pitch, relative to gravity direction	Accelerometer, y-axis, mean	Accelerometer, mean pitch	Accelerometer, mean yaw	Accelerometer, z-axis, mean	Accelerometer, mean yaw, relative to gravity direction	Accelerometer, mean roll, relative to gravity direction	Accelerometer, z-axis, FFT, 4–5 Hz band	Accelerometer, x-axis, mean

3
2
1

Identification of the most important features for the pocket sensor (accelerometer, gyroscope, barometer) only.

8. Feature importance for the wrist sensor (accelerometer configuration)

Importance rank	1	2	3	4	5	6	7	8	9	10
Kickboard vs. rest	Accelerometer, z-axis, mean	Accelerometer, mean yaw, relative to gravity direction	Accelerometer, z-axis, FFT, 14-15 Hz band	Accelerometer, z-axis, FFT, 13-14 Hz band	Accelerometer, z-axis, FFT, 12-13 Hz band	Accelerometer, y-axis, FFT, 6-7 Hz band	Accelerometer, z-axis, FFT, 11-12 Hz band	Accelerometer, y-axis, FFT, 7-8 Hz band	Accelerometer, z-axis, FFT, 10-11 Hz band power	Accelerometer, z-axis, FFT, 9-10 Hz band
Skateboard vs. rest	Accelerometer, vector norm, z-axis, FFT, correlation	Accelerometer, vector norm, FFT, 2-3 Hz band	Accelerometer, vector norm, skewness	Accelerometer, mean pitch, relative to gravity direction	Accelerometer, kurtosis	Accelerometer, z-axis, FFT, 1-2 Hz band	Accelerometer, y-axis, mean	Accelerometer, vector norm, FFT, 1-2 Hz band	Accelerometer, y-axis, FFT, 2-3 Hz band	Accelerometer, x-axis, FFT, 1-2 Hz band
Push-push-glide vs. rest	Accelerometer, mean roll	Accelerometer, mean pitch, relative to gravity direction	Accelerometer, y-axis, mean	Accelerometer, mean pitch	Accelerometer, mean yaw	Accelerometer, z-axis, mean	Accelerometer, mean yaw, relative to gravity direction	Accelerometer, mean roll, relative to gravity direction	Accelerometer, z-axis, FFT, 4-5 Hz band	Accelerometer, x-axis, mean
Kickboard vs. skateboard	Accelerometer, z-axis, mean	Accelerometer, mean yaw, relative to gravity direction	Accelerometer, z-axis, FFT, 14-15 Hz band	Accelerometer, z-axis, FFT, 13-14 Hz band	Accelerometer, z-axis, FFT, 12-13 Hz band	Accelerometer, z-axis, FFT, 11-12 Hz band	Accelerometer, vector norm, FFT, 14-15 Hz band	Accelerometer, vector norm, FFT, 1-2 Hz band	Accelerometer, z-axis, FFT, 14-15 Hz band	Accelerometer, y-axis, FFT, 7-8 Hz band

3
2
1

Identification of the most important features for the wrist sensor (accelerometer configuration) only.

9. Feature importance for the wrist sensor (accelerometer, gyroscope, barometer)

Importance rank	1	2	3	4	5	6	7	8	9	10
Kickboard vs. rest	Accelerometer, z-axis, mean	Accelerometer, mean yaw, relative to gravity direction	Accelerometer, z-axis, FFT, 14-15 Hz band	Accelerometer, z-axis, FFT, 13-14 Hz band	Accelerometer, z-axis, FFT, 12-13 Hz band	Accelerometer, y-axis, FFT, 6-7 Hz band	Accelerometer, z-axis, FFT, 11-12 Hz band	Accelerometer, y-axis, FFT, 7-8 Hz band	Accelerometer, z-axis, FFT, 10-11 Hz band power	Gyroscope, x-axis, FFT, 14-15 Hz band
Skateboard vs. rest	Accelerometer, vector norm, z-axis, FFT, correlation	Accelerometer, vector norm, FFT, 2-3 Hz band	Accelerometer, vector norm, skewness	Accelerometer, vector norm, gyroscope, y-axis, FFT, correlation	Accelerometer, mean pitch, relative to gravity direction	Accelerometer, kurtosis	Accelerometer, y-axis, mean	Accelerometer, z-axis, FFT, 1-2 Hz band	Accelerometer, vector norm, FFT, 1-2 Hz band	Accelerometer, y-axis, FFT, 2-3 Hz band
Push-push-glide vs. rest	Accelerometer, vector norm, 25th percentile	Accelerometer, y-axis, FFT, 4-5 Hz band	Accelerometer, vector norm, FFT, ratio maximum power on total power	Accelerometer, kurtosis	Accelerometer, vector norm, maximum	Accelerometer, y-axis, FFT, 3-4 Hz band	Accelerometer, vector norm, skewness	Accelerometer, vector norm, FFT, main frequence	Accelerometer, vector norm, FFT, 0-1 Hz band	Accelerometer, x-axis, FFT, 2-3 Hz band
Kickboard vs. skateboard	Accelerometer, mean yaw, relative to gravity direction	Accelerometer, z-axis, mean	Accelerometer, z-axis, FFT, 13-14 Hz band	Accelerometer, z-axis, FFT, 14-15 Hz band	Accelerometer, z-axis, FFT, 12-13 Hz band	Accelerometer, vector norm, FFT, 14-15 Hz band	Gyroscope, y-axis, FFT, 1-2 Hz band	Accelerometer, vector norm, FFT, 2-3 Hz band	Accelerometer, vector norm, FFT, 1-2 Hz band	Accelerometer, y-axis, FFT, 7-8 Hz band

2
1

Identification of the most important features for the wrist sensor (accelerometer, gyroscope, barometer) only.

10. Codebook for the importance feature

Variable name in the code	Description
corr_fft_AccVectGx	Accelerometer, vector norm, gyroscope, x-axis, FFT, correlation
corr_fft_AccVectGy	Accelerometer, vector norm, gyroscope, y-axis, FFT, correlation
corr_fft_AccVectGz	Accelerometer, vector norm, gyroscope, z-axis, FFT, correlation
corr_fft_VectX	Accelerometer, vector norm, x-axis, FFT, correlation
corr_fft_VectZ	Accelerometer, vector norm, z-axis, FFT, correlation
Gx_fft_powSIMPS14-15	Gyroscope, x-axis, FFT, 14-15 Hz band
Gy_fft_powSIMPS1-2	Gyroscope, y-axis, FFT, 1-2 Hz band
GyroVect_fft_powSIMPS0-1	Gyroscope, vector norm, FFT, 0-1 Hz band
Gz_25per	Gyroscope, z-axis, 25th percentile
Pitch_avg	Accelerometer, mean pitch
QPitch_g	Accelerometer, mean pitch, relative to gravity direction
QPitch_gSD	Accelerometer, pitch standard deviation, relative to gravity direction
QRoll_g	Accelerometer, mean roll, relative to gravity direction
QRoll_gSD	Accelerometer, roll standard deviation, relative to gravity direction
QYaw_g	Accelerometer, mean yaw, relative to gravity direction
QYaw_gSD	Accelerometer, yaw standard deviation, relative to gravity direction
Roll_avg	Accelerometer, mean roll
Vect_25per	Accelerometer, vector norm, 25th percentile
Vect_fft_fmax	Accelerometer, vector norm, FFT, main frequence
Vect_fft_MaxTotRatio	Accelerometer, vector norm, FFT, ratio maximum power on total power
Vect_fft_powSIMPS0-1	Accelerometer, vector norm, FFT, 0-1 Hz band
Vect_fft_powSIMPS1-2	Accelerometer, vector norm, FFT, 1-2 Hz band
Vect_fft_powSIMPS14-15	Accelerometer, vector norm, FFT, 14-15 Hz band
Vect_fft_powSIMPS2-3	Accelerometer, vector norm, FFT, 2-3 Hz band
Vect_kurt	Accelerometer, kurtosis
Vect_max	Accelerometer, vector norm, maximum
Vect_med	Accelerometer, vector norm, median
Vect_skew	Accelerometer, vector norm, skewness
x_acc_fft_powSIMPS0-1	Accelerometer, x-axis, FFT, 0-1 Hz band
x_acc_fft_powSIMPS1-2	Accelerometer, x-axis, FFT, 1-2 Hz band
x_acc_fft_powSIMPS2-3	Accelerometer, x-axis, FFT, 2-3 Hz band
x_mean	Accelerometer, x-axis, mean
y_acc_fft_powSIMPS0-1	Accelerometer, y-axis, FFT, 0-1 Hz band
y_acc_fft_powSIMPS14-15	Accelerometer, z-axis, FFT, 14-15 Hz band
y_acc_fft_powSIMPS2-3	Accelerometer, y-axis, FFT, 2-3 Hz band
y_acc_fft_powSIMPS3-4	Accelerometer, y-axis, FFT, 3-4 Hz band
y_acc_fft_powSIMPS4-5	Accelerometer, y-axis, FFT, 4-5 Hz band
y_acc_fft_powSIMPS5-6	Accelerometer, y-axis, FFT, 5-6 Hz band
y_acc_fft_powSIMPS6-7	Accelerometer, y-axis, FFT, 6-7 Hz band
y_acc_fft_powSIMPS7-8	Accelerometer, y-axis, FFT, 7-8 Hz band

y_mean	Accelerometer, y-axis, mean
Yaw_avg	Accelerometer, mean yaw
Yaw_std	Accelerometer, yaw standard deviation
z_acc_fft_powSIMPS0-1	Accelerometer, z-axis, FFT, 0-1 Hz band
z_acc_fft_powSIMPS10-11	Accelerometer, z-axis, FFT, 10-11 Hz band power
z_acc_fft_powSIMPS11-12	Accelerometer, z-axis, FFT, 11-12 Hz band
z_acc_fft_powSIMPS1-2	Accelerometer, z-axis, FFT, 1-2 Hz band
z_acc_fft_powSIMPS12-13	Accelerometer, z-axis, FFT, 12-13 Hz band
z_acc_fft_powSIMPS13-14	Accelerometer, z-axis, FFT, 13-14 Hz band
z_acc_fft_powSIMPS14-15	Accelerometer, z-axis, FFT, 14-15 Hz band
z_acc_fft_powSIMPS4-5	Accelerometer, z-axis, FFT, 4-5 Hz band
z_acc_fft_powSIMPS7-8	Accelerometer, z-axis, FFT, 7-8 Hz band
z_acc_fft_powSIMPS9-10	Accelerometer, z-axis, FFT, 9-10 Hz band
z_mean	Accelerometer, z-axis, mean

11. Visualization for some selected features

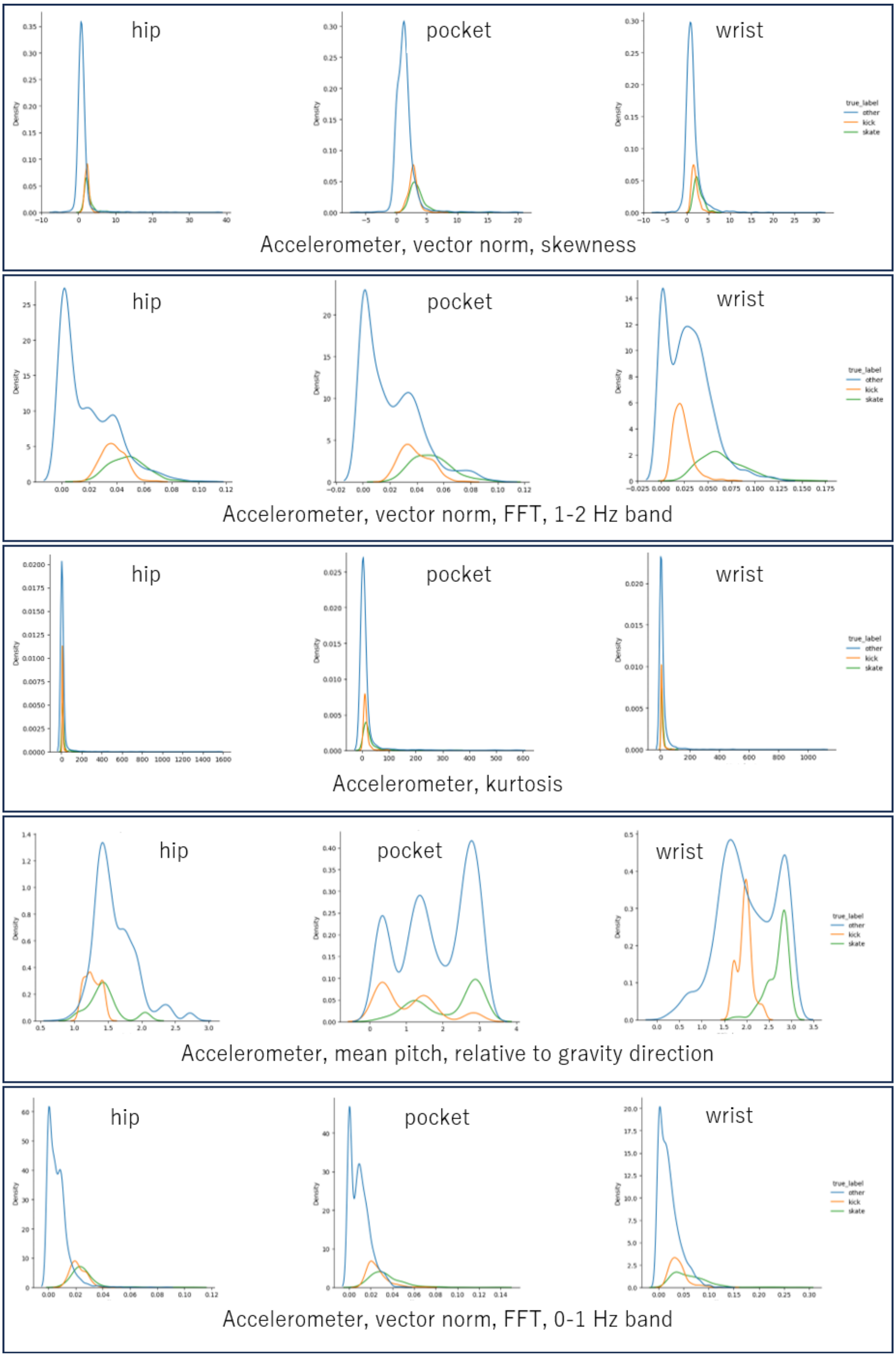

Top 5 singular features (overall; may not be relevant to a specific sensor configuration).

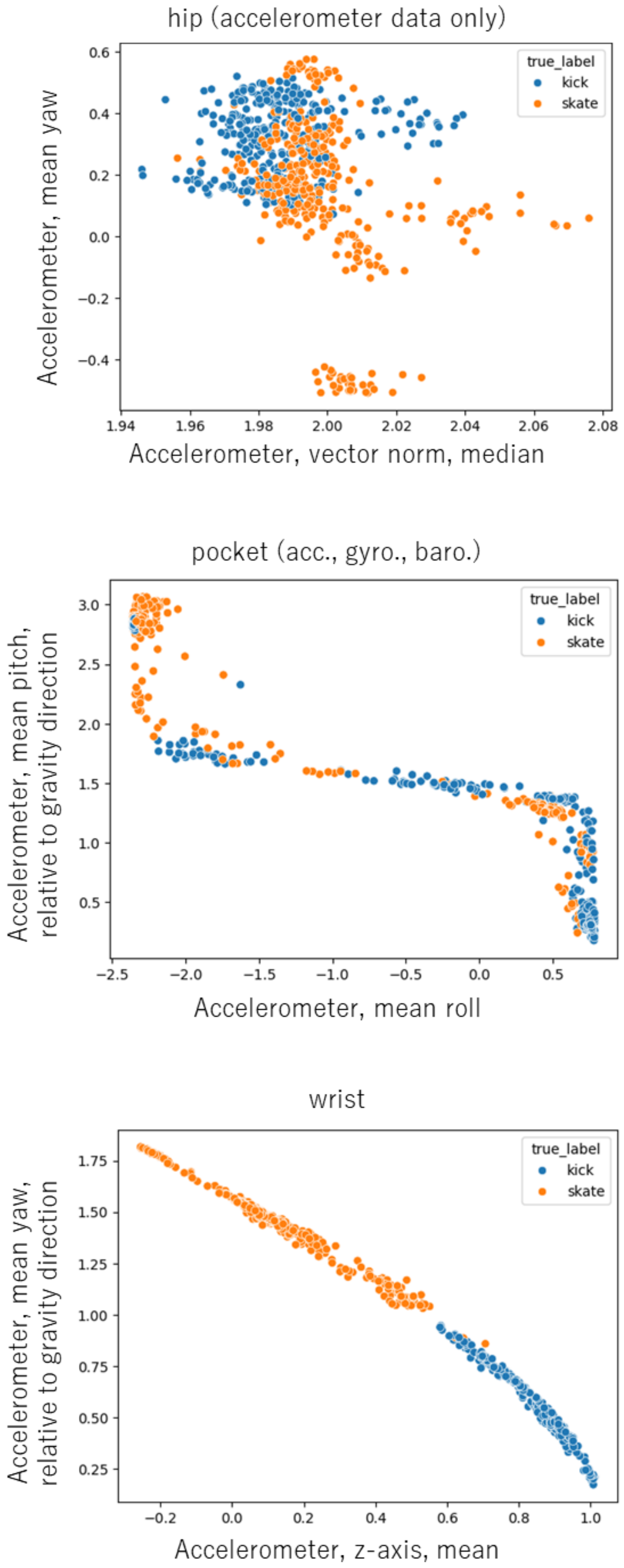

Best feature combinations for kickboard versus kickboard classification. The same top 2 features were found for two wrist sensor configurations

12. Confusion matrices for the kickboard vs. rest analysis removing skateboard data (20-second windows)

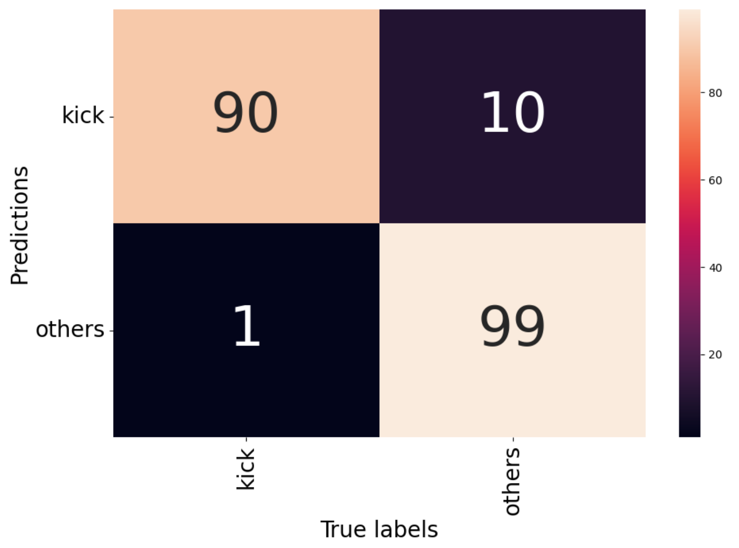

**Hip sensor and accelerometer-derived features
(waist-worn research-grade activity tracker configuration)**

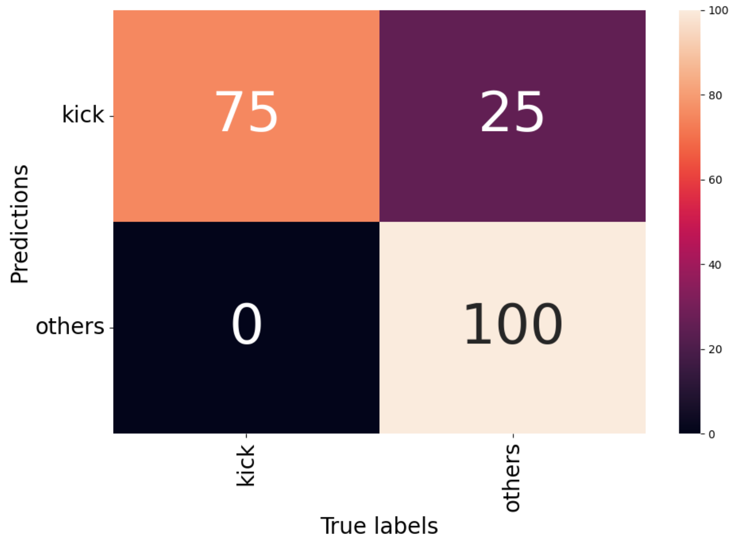

**Pocket sensor and accelerometer-, gyroscope-, and barometer-derived features
(contemporary smartphone device configuration)**

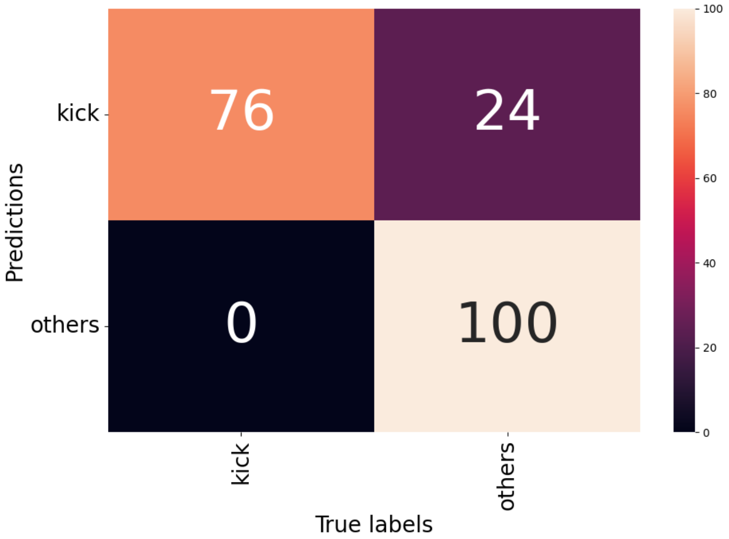

Wrist sensor and accelerometer-derived features (entry level wrist-worn activity tracker configuration)

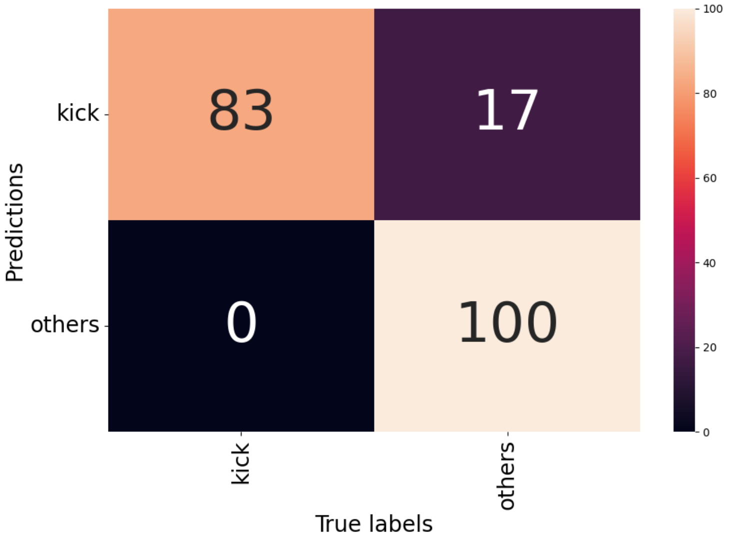

Wrist sensor and accelerometer-, gyroscope-, and barometer-derived features (high-end wrist-worn activity tracker configuration)

13. Confusion matrices for the skateboard vs. rest analysis removing kickboard data (20-second windows)

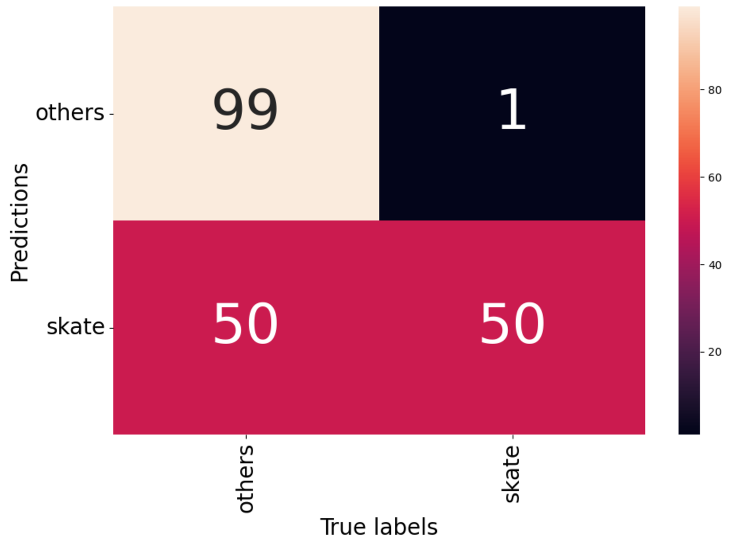

Hip sensor and accelerometer-derived features
(waist-worn research-grade activity tracker configuration)

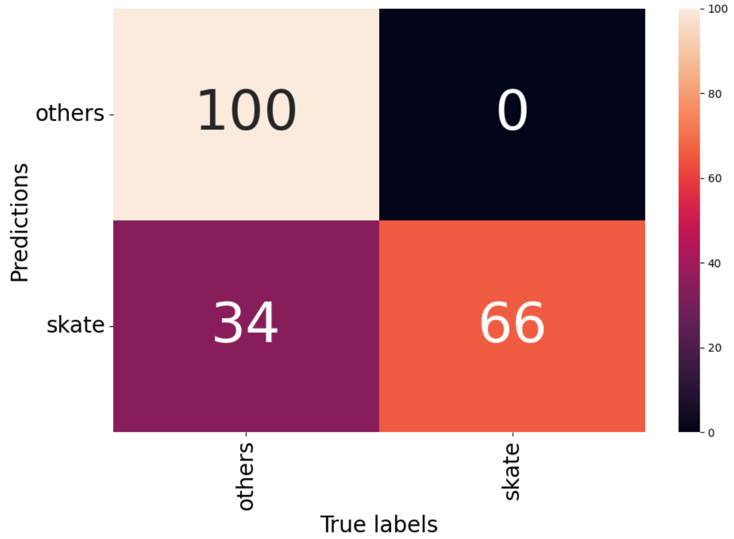

Pocket sensor and accelerometer-, gyroscope-, and barometer-derived features
(contemporary smartphone device configuration)

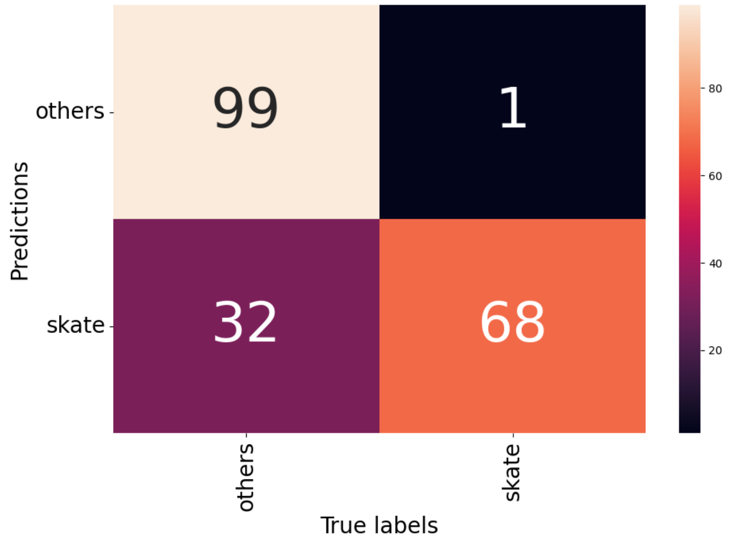

Wrist sensor and accelerometer-derived features (entry level wrist-worn activity tracker configuration)

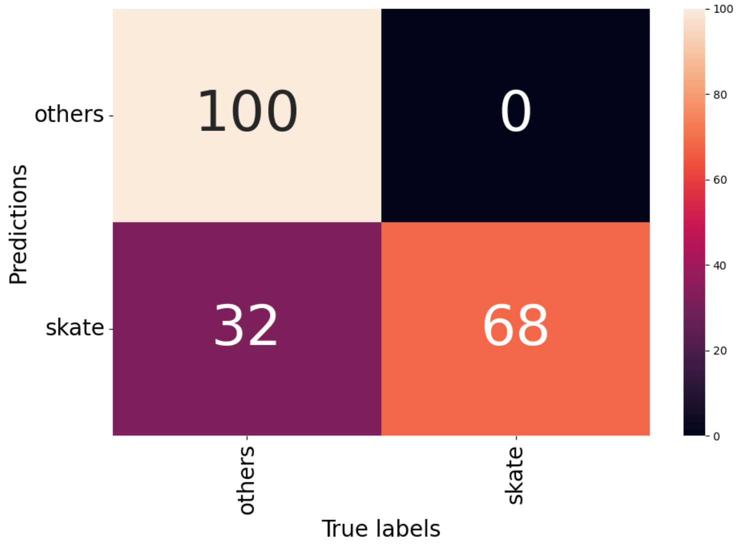

Wrist sensor and accelerometer-, gyroscope-, and barometer-derived features (high-end wrist-worn activity tracker configuration)
